# Prehospital Transdermal Glyceryl Trinitrate for Ultra-Acute Intracerebral Hemorrhage: Data From the RIGHT-2 Trial

**DOI:** 10.1161/STROKEAHA.119.026389

**Published:** 2019-10-07

**Authors:** Philip M. Bath, Lisa J. Woodhouse, Kailash Krishnan, Jason P. Appleton, Craig S. Anderson, Eivind Berge, Lesley Cala, Mark Dixon, Timothy J. England, Peter J. Godolphin, Trish Hepburn, Grant Mair, Alan A. Montgomery, Stephen J. Phillips, John Potter, Chris I. Price, Marc Randall, Thompson G. Robinson, Christine Roffe, Peter M. Rothwell, Else C. Sandset, Nerses Sanossian, Jeffrey L. Saver, A. Niroshan Siriwardena, Graham Venables, Joanna M. Wardlaw, Nikola Sprigg

**Affiliations:** 1 From the Stroke Trials Unit, Division of Clinical Neuroscience, University of Nottingham, United Kingdom (P.M.B., L.J.W., J.P.A., M.D., N.S.); 2 Stroke, Nottingham University Hospitals National Health Service (NHS) Trust, City Hospital Campus, United Kingdom (P.M.B., K.K., N.S.); 3 The George Institute for Global Health, Faculty of Medicine, University of New South Wales, Sydney, Australia (C.S.A.); 4 The George Institute China at Peking University Health Science Center, Beijing, China (C.S.A.); 5 Neurology Department, Royal Prince Alfred Hospital, Sydney Health Partners, NSW, Australia (C.S.A.); 6 Department of Internal Medicine (E.B., A.N.S), Oslo University Hospital, Norway; 7 Department of Neurology (E.C.S.), Oslo University Hospital, Norway; 8 Faculty of Health and Medical Sciences, University of Western Australia (L.C.); 9 East Midlands Ambulance Service NHS Trust, Nottingham, United Kingdom (M.D.); 10 Vascular Medicine, Division of Medical Sciences, GEM, Royal Derby Hospital Centre (T.J.E.), University of Nottingham, United Kingdom; 11 Nottingham Clinical Trials Unit, Queen’s Medical Centre (P.J.G., T.H., A.A.M.), University of Nottingham, United Kingdom; 12 Centre for Clinical Brain Sciences, Edinburgh Imaging and UK Dementia Research Institute at the University of Edinburgh, Chancellor’s Building (G.M., J.M.W.); 13 Department of Medicine, Dalhousie University and Queen Elizabeth II Health Sciences Centre, Halifax, Canada (S.J.P.); 14 Bob Champion Research and Education Building, University of East Anglia, Norwich, United Kingdom (J.P.); 15 Institute of Neuroscience, Newcastle University, United Kingdom (C.I.P.); 16 Department of Neurology, Leeds Teaching Hospitals NHS Trust, United Kingdom (M.R.); 17 Department of Cardiovascular Sciences and NIHR Leicester Biomedical Research Centre, University of Leicester, United Kingdom (T.G.R.); 18 Stroke Research in Stoke, Institute for Science and Technology in Medicine, Keele University, Stoke-on-Trent, United Kingdom (C.R.); 19 Nuffield Department of Clinical Neurosciences, John Radcliffe Hospital, Oxford, United Kingdom (P.M.R.); 20 Research and Development, The Norwegian Air Ambulance Foundation, Oslo, Norway (E.C.S.); 21 Department of Neurology, University of Southern California Keck School of Medicine, Los Angeles (N.S.); 22 Department of Neurology and Comprehensive Stroke Center, David Geffen School of Medicine at UCLA (J.L.S.); 23 Community and Health Research Unit, University of Lincoln, United Kingdom (A.N.S.); 24 Department of Neurology, Royal Hallamshire Hospital, Sheffield, United Kingdom (G.V.).

**Keywords:** allied health personnel, ambulances, blood pressure, humans, nitroglycerin

## Abstract

Pilot trials suggest that glyceryl trinitrate (GTN; nitroglycerin) may improve outcome when administered early after stroke onset.

**Methods—:**

We undertook a multicentre, paramedic-delivered, ambulance-based, prospective randomized, sham-controlled, blinded-end point trial in adults with presumed stroke within 4 hours of ictus. Participants received transdermal GTN (5 mg) or a sham dressing (1:1) in the ambulance and then daily for three days in hospital. The primary outcome was the 7-level modified Rankin Scale at 90 days assessed by central telephone treatment-blinded follow-up. This prespecified subgroup analysis focuses on participants with an intracerebral hemorrhage as their index event. Analyses are intention-to-treat.

**Results—:**

Of 1149 participants with presumed stroke, 145 (13%; GTN, 74; sham, 71) had an intracerebral hemorrhage: time from onset to randomization median, 74 minutes (interquartile range, 45–110). By admission to hospital, blood pressure tended to be lower with GTN as compared with sham: mean, 4.4/3.5 mm Hg. The modified Rankin Scale score at 90 days was nonsignificantly higher in the GTN group: adjusted common odds ratio for poor outcome, 1.87 (95% CI, 0.98–3.57). A prespecified global analysis of 5 clinical outcomes (dependency, disability, cognition, quality of life, and mood) was worse with GTN; Mann-Whitney difference, 0.18 (95% CI, 0.01–0.35; Wei-Lachin test). GTN was associated with larger hematoma and growth, and more mass effect and midline shift on neuroimaging, and altered use of hospital resources. Death in hospital but not at day 90 was increased with GTN. There were no significant between-group differences in serious adverse events.

**Conclusions—:**

Prehospital treatment with GTN worsened outcomes in patients with intracerebral hemorrhage. Since these results could relate to the play of chance, confounding, or a true effect of GTN, further randomized evidence on the use of vasodilators in ultra-acute intracerebral hemorrhage is needed.

**Clinical Trial Registration—:**

URL: http://www.controlled-trials.com. Unique identifier: ISRCTN26986053.

## Abstract 2

Supplemental Digital Content is available in the text.

High blood pressure (BP) is common after the onset of acute intracerebral hemorrhage (ICH) and predicts a poor outcome, in part, by contributing to hematoma expansion.^1–5^ However, trials of BP lowering in acute ICH have had conflicting results: INTERACT-2 (Second Intensive Blood Pressure Reduction in Acute Cerebral Haemorrhage Trial) was marginally positive,^6^ whereas INTERACT-1, the ICH subgroup of the ENOS trial (Efficacy of Nitric Oxide in Stroke), and the ATACH-2 (Second Antihypertensive Treatment of Acute Cerebral Haemorrhage) were neutral,^7–9^ and a subgroup analysis of the SCAST (Scandinavian Candesartan Acute Stroke) trial in ICH was negative.^10^ Consequently, the management of high BP in ICH remains unclear,^11^ and guidelines diverge in their recommendations over target levels of BP control.^12–15^

Glyceryl trinitrate (GTN)—a NO donor—lowered BP by systolic, 7.5/diastolic, 4.2 mm Hg in the ENOS ICH subgroup, as compared with control.^8^ In a meta-analysis and systematic review of individual patient data from 2 randomized trials, GTN was associated with improved functional outcome on the modified Rankin Scale (mRS) in 312 patients with ischemic stroke or ICH who were treated within 6 hours of the onset of symptoms.^16^ These findings led to conduct of the main phase RIGHT-2 (Rapid Intervention With Glyceryl Trinitrate in Hypertensive Stroke Trial), which assessed the effect of prehospital GTN in 1149 patients with ultra-early presumed acute stroke.^17^ However, in patients with a stroke or transient ischemic attack who were recruited within 4 hours (with median time from the onset of symptoms to randomization of 72 minutes), GTN was associated with a nonsignificant worsening of mRS.^17^ Here, we present a detailed prespecified subgroup analysis of the effect of GTN in RIGHT-2 patients with confirmed ICH.

## Methods

### Study Design and Study Population

RIGHT-2 was preregistered as ISRCTN26986053 on 5 March 2015. The trial opened to recruitment in September 2015, and the first participant was recruited on October 22, 2015. Individual participant data will be shared with the Blood Pressure in Acute Stroke Collaboration and Virtual International Stroke Trials Archive; additional information is given in the online-only Data Supplement to this article. RIGHT-2 was a prospective multicenter paramedic-delivered ambulance-based sham-controlled participant- and outcome-blinded randomized controlled trial in adults with ultra-early presumed stroke in the United Kingdom.^17,18^ Briefly, adult patients were eligible for inclusion following an emergency 999 telephone call for presumed stroke if they presented within 4 hours of onset of their symptoms to a trial-trained paramedic from a participating ambulance service and could be taken to a participating hospital. Patients had to have a Face-Arm-Speech-Time (FAST) score of 2 or 3 and a systolic BP ≥120 mm Hg. Patients from a nursing home, or with reduced consciousness (Glasgow Coma Scale <8/15), hypoglycemia (capillary glucose <2.5 mmol/L), or a witnessed seizure were excluded. Detailed inclusion and exclusion criteria are given in the online-only Data Supplement of the main trial publication.^17^ Additional information on the methods is given in the online-only Data Supplement to this article.

### Treatment

Patients were randomly assigned, in 1:1 ratio, to receive transdermal GTN (nitroglycerin; 5 mg as Transiderm-Nitro 5; Novartis, Frimley UK) or sham (DuoDERM hydrocolloid dressing; Convatec, Flintshire United Kingdom). The first treatment (GTN or sham) was administered by the paramedic immediately after randomization in the ambulance, and further treatments were given for ≤3 days while in hospital.

### Outcome Measures

The primary outcome was functional outcome assessed with the 7-level mRS measured at 90 days post-randomization. Outcomes were recorded centrally by telephone by a trained assessor masked to treatment allocation, who used a structured questionnaire to ensure reliable scoring.^19^ This information was collected from a relative or carer if the participant was aphasic or for some other reason incapable of providing the information. If the participant/relative/carer could not be contacted by telephone, a questionnaire covering the same outcome measures was sent by post.

Participants were seen at day 4 (or at hospital discharge, if earlier) to determine adherence to treatment and assess neurological deterioration. Also recorded were the date of discharge from hospital, duration of stay, and discharge destination (to another hospital, institution, or home). Prespecified secondary clinical outcomes at day 90 included activities of daily living (Barthel index), cognition (modified telephone Mini-Mental State Examination; Telephone Interview for Cognition Scale-modified), categorical verbal fluency using animal naming, health-related quality of life (European Quality of Life 5-dimensional 3 level, from which a health status utility value was calculated; European Quality of Life visual analogue scale), and mood (abbreviated Zung depression score), all as used in ENOS and described in the published protocol.^18,20^ Home time was calculated as the number of days between discharge and day 90.

Safety outcomes included all-cause and cause-specific death, investigator-reported hypotension or hypertension occurring during the first 4 days, and serious adverse events (all up to day 5, and fatal thereafter to day 90). Serious adverse events were validated and categorized by expert adjudicators who were blinded to treatment assignment.

### Neuroimaging Outcomes

Nonenhanced brain scans (computed tomography or magnetic resonance imaging) performed on arrival at hospital were collected for central adjudication by expert neuroradiologists masked to treatment assignment, symptoms, and follow-up imaging, using assessments updated from IST-3 (Third International Stroke Trial) and ENOS.^20,21^ Computed tomography/magnetic resonance angiography was also performed in some centers according to local policy and adjudicated centrally. On the next day, a further computed tomography or magnetic resonance scan was performed to assess safety.

### Statistical Analysis

Analyses followed the statistical analysis plan for the overall trial.^22^ The primary outcome (shift on 7-level mRS) was assessed using ordinal logistic regression with adjustment for age, sex, premorbid mRS, baseline FAST score, systolic BP, and time from the onset of symptoms to randomization.^22^ The assumption of proportional odds was tested using the likelihood ratio test. We also performed unadjusted, per-protocol, and imputed (missing mRS data estimated using multiple regression-based imputation) sensitivity analyses for completeness. Heterogeneity of the treatment effect on the primary outcome was assessed for the purpose of hypothesis generation in prespecified subgroups by adding an interaction term to an adjusted ordinal logistic regression model. Death was analyzed using adjusted Cox regression models. Other outcomes were assessed using adjusted binary logistic regression, Cox regression, ordinal logistic regression, multiple linear regression, and ANCOVA (BP). A prespecified global outcome (comprising ordered categorical or continuous data for mRS, Barthel index, Zung depression score, Telephone Interview for Cognition Scale-modified, and European Quality of Life 5-dimensional health status utility value) was analyzed using the Wei-Lachin test.^23^ Participants who did not receive their assigned treatment or did not adhere to the protocol, or who had a stroke mimic, were all still followed up in full at day 90 and are included in the main analyses.

## Results

### Demographics

**Figure 1.**
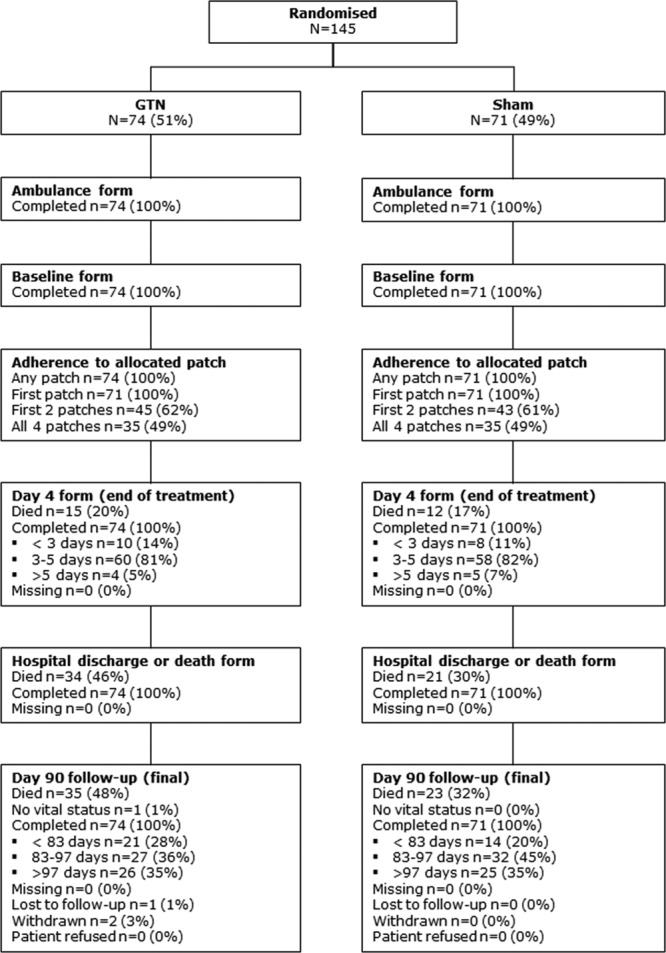
CONSORT (Consolidated Standards of Reporting Trials) diagram in participants with a final diagnosis of intracerebral hemorrhage. GTN indicates glyceryl trinitrate.

Of the 1149 recruited patients, 145 (13%; GTN, 74; sham, 71) had a final hospital diagnosis of ICH based on clinical presentation and neuroimaging (Figure 1). Characteristics at baseline were well balanced between GTN and sham (Table 1): mean age, 73 (SD, 13) years; women, 64 (44%); FAST score, 3111 (77%); time from onset to randomization median, 74 (interquartile range, 45–110) minutes. Forty-one (44%) participants were taking an antithrombotic drug before their stroke. Adherence was excellent with 100% of participants receiving the first randomized treatment (Table I in the online-only Data Supplement).

**Table 1.**
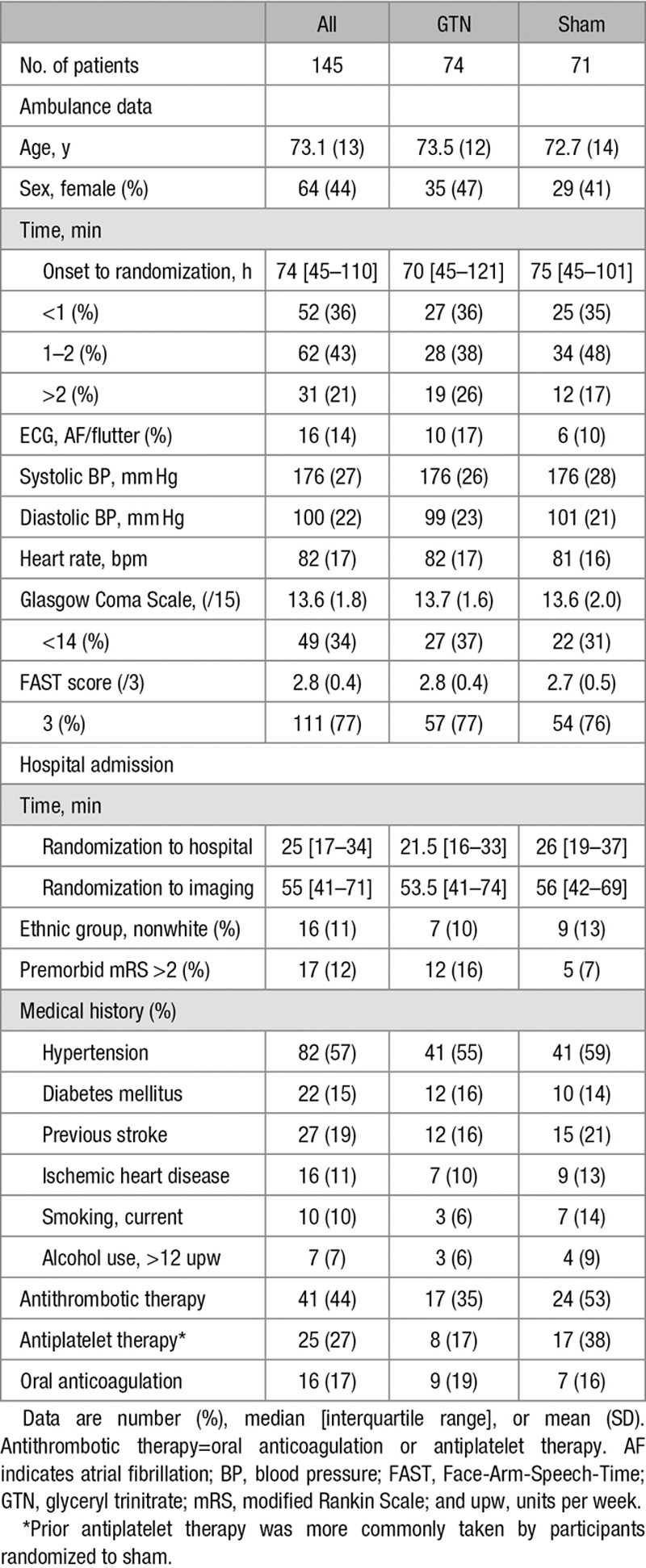
Baseline Characteristics

### Clinical Outcomes

**Figure 2.**
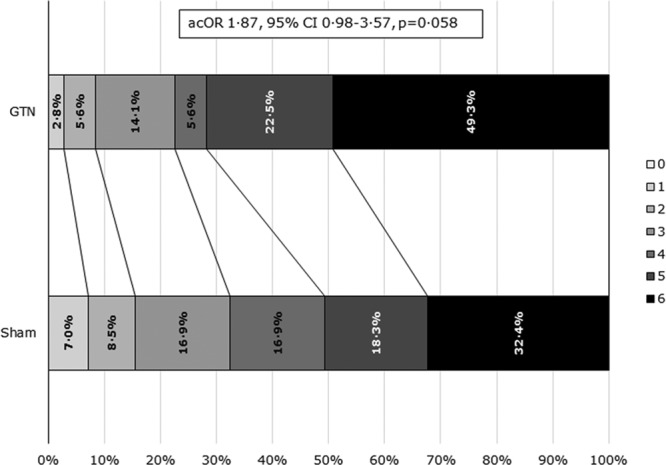
Shift in modified Rankin Scale in 145 participants with a final diagnosis of intracerebral hemorrhage by treatment group. Comparison by ordinal logistic regression with adjustment for age, sex, premorbid modified Rankin Scale, face-arm-speech time test, pretreatment systolic blood pressure, and time to randomization. The effect of treatment for glyceryl trinitrate (GTN) vs sham is shown as adjusted common odds ratio (acOR).

**Figure 3.**
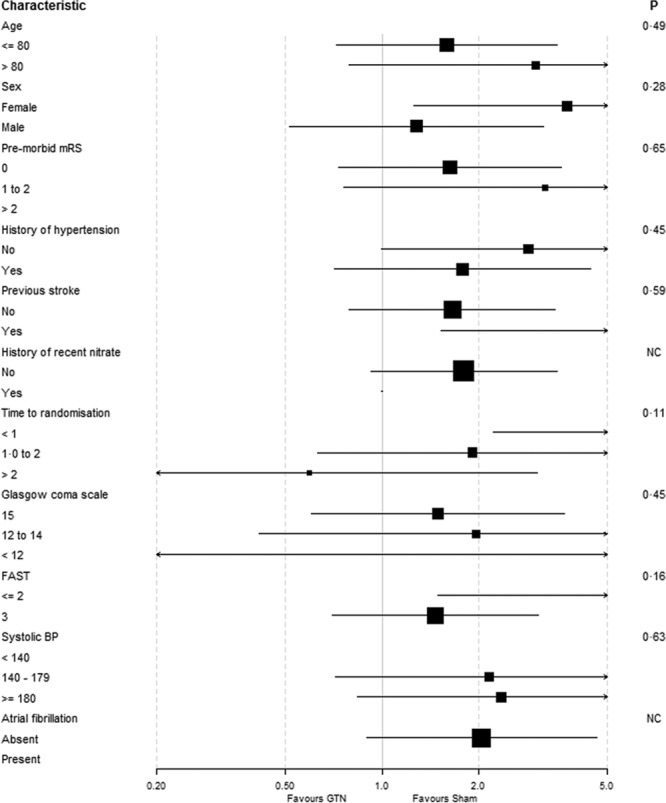
Forest plot showing modified Rankin Scale (mRS) in prespecified subgroups of participants with intracerebral hemorrhage, with *P* value for interaction. Heterogeneity of the treatment effect on the primary outcome was assessed by adding an interaction term to an ordinal logistic regression model with adjustment as in Figure 2. BP indicates blood pressure; FAST, Face-Arm-Speech-Time; and GTN, glyceryl trinitrate.

After treatment, systolic and diastolic BP were nonsignificantly lower in the GTN group by mean 4.4/3.5 mm Hg at hospital admission, as compared with sham (Figure I in the online-only Data Supplement). One hundred forty-two (98%) of participants with ICH had the primary outcome (mRS) measured at 3 months (Table 2). We found some evidence, albeit statistically nonsignificant, that GTN was associated with a worse functional outcome, GTN median 5 (interquartile range, 4–6) versus sham median 5 (interquartile range, 3–6; adjusted common odds ratio [OR], 1.87; 95% CI, 0.98–3.57; Figure 2). In 4 planned sensitivity analyses of the primary outcome,^17^ all comparisons were significant statistically with a worse mRS in the GTN as compared with sham group (Table 2). When assessing interactions of the effect of GTN on mRS by prespecified subgroups, a nonsignificant effect of time to randomization was apparent with participants recruited within 1 hour of symptom onset faring much worse with GTN (Figure 3).

**Table 2.**
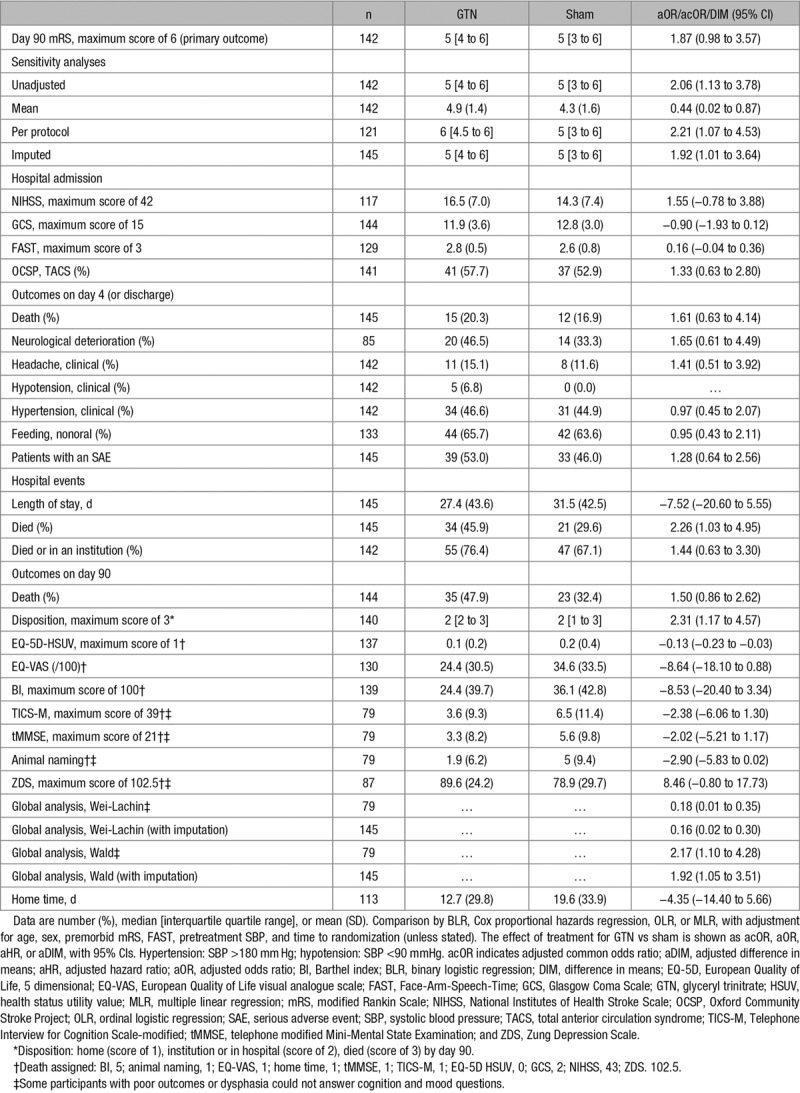
Primary Outcome and Key Secondary Outcomes

After treatment in the ambulance, Glasgow Coma Scale and FAST scores had separated by hospital admission and were nonsignificantly worse in the GTN group as compared with sham (Figure IIA and IIB in the online-only Data Supplement). More than 40% of deaths occurred by day 4; GTN was associated with a significant increase in deaths in hospital and nonsignificant increase by day 90 (Table 2; Figure III in the online-only Data Supplement). GTN was associated with a worse discharge disposition (with more participants going to an institution) and quality of life and possibly more mood disturbance. Global analyses based on all available data (n=79) and following imputation of missing data, and encompassing the original ordinal or continuous data for mRS, Barthel index, Telephone Interview for Cognition Scale-modified, Zung depression score, and European Quality of Life 5-dimensional health status utility value, were significantly worse for GTN (Wei-Lachin test, Mann-Whitney difference, 0.18; 95% CI, 0.01–0.35; Figure IV in the online-only Data Supplement).

### In-Hospital Management and Treatment

While there may have been more use of antihypertensive therapy in the sham group as compared with the GTN group (Table II in the online-only Data Supplement), use of labetalol did not differ between the groups. Although admission to intensive care was uncommon (15%), ventilation was more common in participants randomized to GTN than sham (Table II in the online-only Data Supplement). Conversely, GTN was associated with less physiotherapy and speech therapy.

### Neuroimaging Findings

Admission and day 2 to 4 scans were performed at median 2.3 hours and median 28.9 hours after ICH, respectively (Table III in the online-only Data Supplement). On admission to hospital, GTN was associated with larger hematoma on admission (assessed as maximum length: adjusted common OR, 1.95; 95% CI, 1.07–3.58; Table III in the online-only Data Supplement; Figure V in the online-only Data Supplement) and more mass effect (adjusted common OR, 2.42; 95% CI, 1.26–4.68; Figure VI in the online-only Data Supplement). There was no difference in intraventricular volume. On repeat imaging on day 2, GTN was associated with hematoma that were larger and more irregular in shape and increased perihematomal edema and midline shift (Table III in the online-only Data Supplement).

## Discussion

This prespecified subgroup analysis of the RIGHT-2 trial explored the effect in those patients who were recruited with a final hospital diagnosis of ICH. Considering that the primary analysis for the overall trial population was neutral, ICH patients randomized to GTN had a worse outcome that was apparent across the primary end point and multiple other clinical dimensions covering dependency, quality of life, discharge disposition and a global analysis of these, and measures of hematoma morphology; tendencies to more death and worse disability, cognition, and mood were also present. With matched characteristics at baseline, the negative effect of GTN appeared to start rapidly after patch placement manifesting as a tendency to early separation of Glasgow Coma Scale and FAST scores by 25 minutes; findings of larger hematoma and more mass effect on imaging shortly after hospital admission by 55 minutes; altered hospital activity with patients randomized to GTN needing more ventilation, less physiotherapy and speech therapy, and increased death in hospital; and then worse outcome at 90 days.

The finding that GTN appears to worsen outcome in ICH when administered in the ultra-acute period after stroke was unexpected since a meta-analysis of data from 2 trials suggested that GTN improved outcome when administered to patients with ICH within 6 hours of onset.^16^ A key difference between these trials is that the median time from ICH onset to randomization was 74 minutes in RIGHT-2 versus 280 minutes in ENOS early ICH.^24^ In the overall stroke/transient ischemic attack population in RIGHT-2, there was a profound time-by-treatment interaction with a negative effect of GTN on mRS in patients randomized within 1 hour and a tendency to benefit in those randomized beyond 2 hours. Although this time-by-treatment interaction was not significant in the ICH subgroup alone, GTN worsened outcome when randomized within 1 hour (OR, 8.39; 95% CI, 2.22–31.69) and yet was associated with a positive tendency beyond 2 hours (Figure 3). Further, RIGHT-2 participants had more premorbid dependency, an increased prevalence of baseline background imaging changes, and hematoma that were more than twice the size observed in ENOS (Table III in the online-only Data Supplement). Finally, treatment was given for 7 days in ENOS and only 4 days in RIGHT-2. These differences may explain the variation in overall outcome and response to GTN seen between RIGHT/ENOS early and RIGHT-2. Potential mechanisms for why GTN worsens outcome if given early after ICH include inhibiting the first (vasoconstriction) and second (platelet plugging) phases of hemostasis, as detailed in the online-only Data Supplement. This time-dependent pattern of negative-neutral-positive-neutral findings is novel and contrasts with reperfusion therapies that exhibit a positive-neutral time course. Hence, it could be hypothesized that nonreperfusion therapies should not be started in the ultra-acute period, perhaps reflecting that the early stunned and fragile brain does not respond well to active modulation by external interventions.

The subgroup analysis presented here has several strengths. First, it was prespecified and follows on from previous work suggesting that GTN would improve outcome in the hyperacute phase of stroke, including in ICH.^16^ Second, RIGHT-2 had limited exclusion criteria; so the results probably apply to most patients with prehospital ICH. Third, participants were masked to treatment and unaware of treatment assignment at day 90. Last, the results show clear internal validity with parallel negative effects of GTN seen on clinical, radiological, and hospital activity measures.

Similarly, there are several limitations. First, the ICH sample in RIGHT-2 was small (n=145) although this was driven by the overall sample size of the trial (n=1149) and the proportion of these with ICH (n=13%). In reality, the proportion of ICH of overall stroke was high at 24% and larger than seen in hospital-based trials reflecting the relatively unselected nature of the study. Second, technically, this substudy is neutral since the CIs for the result of the analysis of the primary outcome analysis (ordinal shift in mRS) narrowly crossed OR of 1.00. The small sample size and technical neutral result raise the possibility that the results reflect the play of chance or systematic confounding. Chance is unlikely in the presence of internally consistent results across clinical, imaging, and hospital activity findings. Systematic confounding, due perhaps to imbalances in unmeasured demographic, clinical, or imaging variables at baseline, is possible and highlights the challenges of adequately describing participants in the time-limited prehospital ambulance environment.

In summary, we have shown that the ultra-acute use of GTN may be harmful in patients with ICH and especially within 2 hours. This could result from inhibition of the earliest vasoconstrictory and platelet plugging phases of hemostasis, mechanisms that might apply to other vasodilators. In this respect, RIGHT-2 is the first large trial to test the effect of inhibiting vasoconstriction in this time-critical period after ICH. However, further trials are needed to determine whether the results reflect systematic confounding or a real effect of this agent or BP-lowering treatment more broadly, particularly as vasodilators/antihypertensive agents are widely used in neurocritical care in ICH patients. In the meantime, we recommend that GTN (and probably other nitrovasodilators) should not be used in the prehospital setting in patients with possible stroke outside of a randomized controlled trial.

## Acknowledgments

We thank the patients who participated in this trial and their relatives, the clinical and research teams of the various ambulance services and hospitals, and the paramedics who recruited and treated the patients. We acknowledge support of the English National Institute for Health Research (NIHR) Clinical Research Network and that the coordination between multiple ambulance services and hospitals and large recruitment would not have been possible without NIHR network support. A complete list of the RIGHT-2 (Rapid Intervention With Glyceryl Trinitrate in Hypertensive Stroke Trial-2) investigators is provided in the primary publication.^17^

## Sources of Funding

This work was supported by the British Heart Foundation (grant No. CS/14/4/30972).

## Disclosures

P.M. Bath is Stroke Association Professor of Stroke Medicine and is a National Institute Health Research (NIHR) Senior Investigator. He reports grants from British Heart Foundation (BHF) during the conduct of the study and personal fees and other fees from Sanofi, Nestlé, DiaMedica, Moleac, Platelet Solutions, Phagenesis, and ReNeuron, outside the submitted work. J.P. Appleton was funded, in part, by the BHF during the conduct of the study. Dr Anderson reports grants from the National Health and Medical Research Council of Australia and Takeda and personal fees from Takeda, Amgen, and Boehringer Ingelheim outside of the submitted work. M. Dixon was funded by the BHF during the conduct of the study. T.J. England and Dr Montgomery report grants from BHF during the conduct of the study. Dr Mair is supported by National Health Service (NHS) Lothian Research and Development Office and reports grants from The Stroke Association (TSA) and The Royal College of Radiologists. C.I. Price reports grants from Nottingham University and BHF during the conduct of the study. Dr Robinson is an NIHR Senior Investigator and reports grants from BHF during the conduct of the study. Dr Roffe reports grants from NIHR Health Technology Assessment during the conduct of the study; personal fees from Allergan, Air Liquide, Merz, Boehringer, Bayer, Johnson & Johnson, Sanofi, and Emtensor; nonfinancial support from European Stroke Conference and Trident; and other support from Firstkind Medical, Medtronic, and Brainomix outside the submitted work. P.M. Rothwell reports grants from Wellcome Trust during the conduct of the study. Dr Sandset reports personal fees from Novartis and Bayer outside of the submitted work. J.M. Wardlaw reports grants from the BHF during the conduct of the study and grants from Medical Research Council, Chief Scientist Office, Leducq, EU H2020, TSA, BHF, and Alzheimer’s Society outside the submitted work. N. Sprigg reports grants from BHF and Research Councils UK (RCUK), during the conduct of the study. The other authors report no conflicts.

## Supplementary Material
