# Supplementary material for: Prehospital Transdermal Glyceryl Trinitrate for Ultra-Acute Intracerebral Hemorrhage: Data From the RIGHT-2 Trial
Source: Stroke. 2019 Oct 7;50(11):3064–71. doi: 10.1161/STROKEAHA.119.026389 (PMC6824503; doi:10.1161/STROKEAHA.119.026389)
Supplement: Supplementary file 1 [file str-50-3064-s001.pdf]

SUPPLEMENTAL MATERIAL

Pre-hospital transdermal glyceryl trinitrate for ultra-acute intracerebral haemorrhage- data from the RIGHT-2 trial

Writing Committee
Philip M Bath, Lisa J Woodhouse, Kailash Krishnan, Jason P Appleton, Craig S Anderson, Eivind Berge, Lesley Cala, Mark Dixon, Timothy J England, Peter J Godolphin, Trish Hepburn, Grant Mair, Alan A Montgomery, Stephen J Phillips, John Potter, Chris Price, Marc Randall, Thompson G Robinson, Christine Roffe, Peter M Rothwell, Else C Sandset, Nerses Sanossian, Jeffrey L Saver, A Niroshan Siriwardena, Graham Venables, Joanna M Wardlaw, Nikola Sprigg

TABLE OF CONTENTS

	Page
Title	1
Writing Committee	1
Supplemental Methods	2
Supplemental Results	4
Supplemental Discussion	6
Contributors	8
List of supplementary Tables and Figures	10
Supplemental Tables (start from)	11
Supplemental Figures (start from)	19
Supplemental References	27

SUPPLEMENTAL METHODS (additional information)

Study design and study population

- No ambulances had a CT scanner to provide immediate diagnosis before hospital.
- The study was approved by the UK regulator (Medicines and Healthcare products Regulatory Agency, reference: 03057/0064/001–0001; Eudract 2015–000115–40) and national research ethics committee (IRAS: 167115) and was adopted by the National Institute for Health Research Clinical Research Network.

Randomisation and consent

- Randomisation was stratified by ambulance station with blocks of 4 packs (2 active, 2 control) in random permuted order.
- Paramedics managed the primary consent process, and patients with capacity gave written informed consent that covered the whole trial. If capacity was absent, proxy consent was obtained from an accompanying relative, carer or friend if present, or from the paramedic if no accompanying person was present. Participants who regained capacity were then given the opportunity to consent in hospital.
- The final diagnosis was made after arrival at hospital by the principal investigator based on clinical and neuroimaging findings and was categorised as ICH, ischaemic stroke, TIA, or non-stroke/TIA mimic.

Outcome measures

- The primary analysis involved a comparison of the distribution of all 7 levels of the mRS ('shift analysis') between the treatment groups.
- Neurological deterioration was defined as an increase in the National Institutes of Health stroke scale, NIHSS ≥4 points from hospital admission to day 4 or worsening conscious level in the NIHSS consciousness domain item Ia.

Neuroimaging outcomes (additional information)

- Imaging outcomes on admission to hospital included the presence of pre-stroke features (atrophy, periventricular lucencies, old strokes, frailty score and small vessel disease score), haematoma location, size (categorised maximum diameter), volume,[1] extension (to subarachnoid spaces or ventricles), mass effect including midline shift (assessed on a validated 7-point scale),[2] and qualitative measures suggestive of active acute bleeding (e.g. hypodensity sign, island sign and swirl sign,[3] and spot sign if CT angiography was performed [4]).
- A further CT or MR scan was performed on day 2 to assess safety; the same factors were assessed blind to treatment assignment and baseline scan findings.

Sample size

- We estimated that a total sample size of 850 participants (425 in each arm) was required to detect a shift in mRS with common odds ratio 0.70,[5] significance level 5%, power 90%, assumed distribution of mRS scores as shown in the appendix of the main publication,[6] loss to follow-up 3%, joint mimic and TIA rate of 20%, and reduction for baseline co-variate adjustment 20%.[7] We assumed that approximately 15% of participants would have a final diagnosis of ICH.[8]
- The sample size was determined for the whole trial and not for this or any other subgroup of patients.
- During the trial, the non-stroke diagnosis rate was found to exceed 30%, and the sample size was increased from 850 to 1050 to maintain statistical power.

Statistical analysis (additional information)

- A *post hoc* global analysis based on dichotomous outcomes (mRS >2, BI <30, TICS-M <5, ZDS >80, HSUV <0.2) was performed as in the NINDS alteplase trial.[9]
- No adjustments are made for multiplicity of testing since all secondary analyses were hypothesis-generating and designed to support the primary analysis.
- Data are shown as number (%), median [interquartile range, IQR], mean (standard deviation, SD), and odds ratio, hazard ratio, difference in means or Mann-Whitney difference (test of global outcome) with 95% confidence intervals (CI).
- Analyses were done as randomised in participants with ICH and with observed outcome data, and performed with SAS software (version 9.3).

Data sharing

- Individual participant data will be shared with the Blood pressure in Acute Stroke Collaboration (BASC) and Virtual International Stroke Trials Archive (VISTA).
- From Jan 1, 2021, the Chief Investigator (with approval from the Trial Steering Committee as necessary) will consider other requests to share individual participant data via email at: right-2@nottingham.ac.uk. We will require a protocol detailing hypothesis, aims, analyses, and intended tables and figures. Where possible, we will perform the analyses; alternatively, de-identified data and a data dictionary will be supplied for the necessary variables for remote analysis. Any sharing will be subject to a signed data access agreement.
- Ultimately, the entire trial dataset will be published.

SUPPLEMENTAL RESULTS (additional information)

Demographics

- 7 (5%), 52 (36%) and 114 (79%) of participants received study drug within 30, 60 and 120 minutes of symptom onset, respectively (Table 1).
- Although adherence was excellent with 100% of participants receiving the first randomised treatment, adherence on days 2, 3 and 4 was considerably lower at 61%, 59% and 55% respectively (Supplemental Table I).
- At baseline in the ambulance, blood pressure was mean 176 (SD 27)/100 (SD 22) mmHg (Table 1) and fell in both treatment groups over the four days after randomisation (Supplemental Figure I).

Clinical outcomes

- All variables used in adjustment were significantly related to mRS: age ($p<0.001$), sex ($p=0.042$), premorbid mRS ($p=0.0044$), FAST ($p=0.0006$), time ($p=0.0051$), except for SBP ($p=0.79$).
- In *post hoc* analyses:
    - the primary outcome model remained non-significant if the baseline GCS was used rather than FAST for statistical adjustment (acOR 1.94, 95% CI 1.00-3.80);
    - GTN was associated with more participants having a severe functional outcome, mRS >4: 51 (72%) vs 36 (51%) (aOR 2.78, 95% CI 1.20, 6.43);
    - there was no interaction between use of antithrombotic drugs and the effect of GTN on functional outcome;
    - a global outcome based on dichotomous outcomes (Wald test) was significant (OR 2.17, 95% CI 1.10, 4.28);
    - in an analysis suggested by a reviewer, the effect of GTN vs sham on mRS became neutral when haematoma location (lobar, deep, posterior) was added to the primary analysis model (OR 1.30 (95% CI 0.63, 2.71; $p=0.48$);
    - the interaction term between haematoma location, treatment and outcome was significant ($p=0.0076$); whilst GTN was associated with significant hazard in lobar/posterior haemorrhage (OR 5.03, 95% CI 1.60, 15.81; $p=0.0058$) there was no effect on mRS in deep haemorrhages (OR 0.79, 95% CI 0.32, 1.96; $p=0.62$).

In hospital management and treatment

- A majority of participants (69%) received open-label antihypertensive therapy between admission and day 4, typically between 4 and 5 hours after randomisation (Supplemental Table II). This included a variety of antihypertensive drug classes with the most common being ß-receptor antagonists, calcium channel blockers and nitrates. The use of these did not differ between GTN and sham treatment groups.

Neuroimaging findings

- The presence of pre-stroke imaging features did not differ between the treatment groups (Supplemental Table III).
- A majority of haematoma were deep, and location did not differ between the treatment groups.
- In an analysis suggested by a reviewer, the effect of GTN vs sham on peri-haematomal oedema remained significantly increased at day 2 with the addition of haematoma size to the primary analysis model (mean difference 6.87, 95% CI 0.26, 13.48; $p=0.041$);

- There was no interaction between treatment and prior antithrombotic therapy on haematoma size (MD 0.71, 95% CI -0.22, 1.65)
- Haematoma were one-to-three fold larger than in previous ICH trials of pharmacological agents, and approached the size studied in surgical trials (Supplemental Table IV).
- Few CT angiograms were performed, and a positive spot sign was infrequent (12%); this rate did not differ between GTN and sham.

Relationship between imaging variables and outcome

- Although none of the pre-stroke imaging features were associated with haematoma expansion by day 2-4, or death or mRS at day 90 (Supplemental Table VI), the presence of periventricular lucencies, and higher frailty and SVD scores, appeared to either modify or mediate the effect of GTN on outcome.
- Multiple haematoma characteristics at hospital admission were associated with haematoma expansion by day 2-4, and death or mRS at day 90, including the maximum length (Supplemental Figure VII) and volume, and presence of irregular shape and density, island sign, hypodensity sign, swirl sign, intraventricular haemorrhage, mass effect, perihaematomal oedema, midline shift, and hydrocephalus (Supplemental Table V). Of these, haematoma shape, intraventricular volume, presence of hydrocephalus appeared to either modify or mediate the effect of GTN on haematoma expansion or outcome.

Meta-analysis of completed trials

- Addition of the results for participants with ICH in RIGHT-2 to those in RIGHT and ENOS-early resulted in neutral effects for end-of-trial death or dependency (mRS>2), OR 0.98 (95% CI 0.25–3.86) and death, OR 0.55 (95% CI 0.12–2.47) (Supplemental Figure VIII).
- Overall, there was no difference between GTN and sham when data were analysed by time from onset to randomisation for 0-2 hours and 2-6 hours for either outcome although both death or dependency (mRS>2) and death at day 90 were increased in RIGHT-2 participants randomised within two hours of ICH onset (Supplemental Figure VIII).
- Heterogeneity was present for both outcomes (death or dependency I^2 53%; death I^2 70%) and the point estimates were in opposite directions such that very early treatment (0-2 hours) with GTN might be hazardous whilst slightly later treatment (2-6 hours) might be beneficial (Supplemental Figure VIII).

SUPPLEMENTAL DISCUSSION

- Different mechanisms of action may explain the different effects on outcome throughout the ultra-acute, hyper-acute and acute phases of stroke. For example, vasodilators may have negative, neutral or positive effects depending on when they are started over the first 48 hours after ICH, as seen in recent BP-lowering trials (Supplemental Table VI).
- The negative effect apparent in the first 2 hours after ICH (as seen here in RIGHT-2) may reflect attenuation of the first phase of haemostasis based on rapid but transient vasoconstriction;[10] mechanisms for driving vasoconstriction involve:
  - reduced nitric oxide and prostacyclin from damaged endothelium;
  - thromboxane A2 liberated from activated platelets;
  - nervous reflexes from "pain";
  - direct injury to vascular smooth muscles.

  Hence, administration of GTN would oppose reduced endothelial NO synthesis and so attenuate the vasoconstrictory response.
- Additionally, most vasodilators have mild antiplatelet effects, as may be true for GTN,[11] and so prevent the second phase of haemostasis based on platelet-plugging.
- The combination of less vasoconstriction and platelet plugging will lead to increased bleeding.
- The interaction of haematoma irregular shape on the effect of GTN on haematoma expansion and death supports this interpretation since irregular finger-like projections are also a marker of cerebral amyloid angiopathy-associated ICH which is a condition in which micro-vessels have reduced vasomotor activity.[12]
- The subsequent positive effect of vasodilators between 2 and 6 hours as seen in INTERACT-2 and GTN-early-ICH [13, 14] presumably reflects the benefit of lowering BP and so limiting haematoma expansion.
- If treatment is started later than about 6 hours then there is no benefit since haematoma expansion has largely finished.[15]
- If this pattern of effect is correct, then GTN, and potentially other vasodilators, should not be given to lower BP very early, say within 2 hours, after ICH. This may have major implications for the very early management of high BP in ICH.
- It is interesting to note a similar time-dependent effect for another intervention, namely physical therapy for stroke with hazard if started acutely,[16] benefit if started in the sub-acute period, and then no benefit if started weeks or months later.

This subgroup report highlights several findings that may inform future trial design in ICH.

1. A simple measure of haematoma size, namely maximum length, is both prognostic for functional outcome and sensitive to a treatment effect. This measure could also be easily used in clinical practice.
2. It could be argued that patients with large ICH (>5 cm in maximum length) should be excluded from drug-based trials in view of their very poor outcome although how this would be identified in the majority of ambulances without a CT scanner remains unclear at present.
3. Other haematoma characteristics such as shape may be sensitive measures of treatment effect.
4. Pre-hospital treatment can alter hospital treatment activity such as critical care and therapy utilisation; these measures should become secondary outcomes in future ambulance-based trials.
5. A global outcome (based on ordinal or continuous data and analysed using the Wei-Lachin test) may be more sensitive to treatment effects and avoid the need to

identify a single outcome measure; whether such global analysis should comprise the primary outcome of trials needs further testing and discussion.

6. It is questionable whether GCS and FAST offer sufficient granularity in quantifying severity and acting as covariates in adjusted statistical analyses; we note that several alternative pre-hospital scoring systems for severity are in development.

CONTRIBUTORS

- The trial was conceived and designed by the grant applicants, and they wrote the protocol.
- The trial was overseen by a Trial Steering Committee (which included three independent members and a patient-public representative), and advice was given by an International Advisory Committee.
- The day-to-day conduct of the trial was run by a Trial Management Committee, which was based at the Stroke Trials Unit in Nottingham, UK. Study data were collected and quality-assured by the RIGHT-2 Coordinating Centre in Nottingham.
- Analysis, interpretation, and report writing were performed independently of the funder and sponsor.
- PMB was chief investigator, a grant applicant, participated in the Steering Committee, collected, verified, and analysed data and drafted this report, and is project guarantor.
- LJW was trial statistician, involved in the design of the trial, participated in the Steering Committee, and analysed data.
- KK performed brain scan measurements in participants with intracerebral haemorrhage.
- JPA was the trial physician supporting the chief investigator and trial delivery.
- CSA was an international adviser who provided guidance on trial delivery and interpretation.
- EB was an international adviser who provided guidance on trial delivery and interpretation.
- LC adjudicated brain scans.
- MD was the national paramedic lead coordinating ambulance service trial delivery.
- TJE was a grant applicant, participated in the Steering Committee, and advised on trial delivery.
- PJG was statistician to the Data Monitoring Committee.
- TH wrote the approval documents and information sheets and provided statistical advice.
- GM adjudicated brain scans.
- AAM was a statistician, grant applicant, and participated in the Steering Committee.
- SJP was an international adviser who provided guidance on trial delivery and interpretation.
- JP was a grant applicant, participated in the Steering Committee, and advised on trial delivery.
- CIP was a grant applicant, participated in the Steering Committee, and advised on trial delivery by ambulance services.
- MR adjudicated serious adverse events.
- TGR was a grant applicant, participated in the Steering Committee, and advised on trial delivery.
- CR was a grant applicant, participated in the Steering Committee, and advised on trial delivery.
- PMR was a national adviser who provided guidance on trial delivery and interpretation.
- ECS was an international adviser who provided guidance on trial delivery and interpretation.
- NSa was an international adviser who provided guidance on ambulance trial delivery and interpretation.
- JLS was an international adviser who provided guidance on ambulance trial delivery and interpretation.

- ANS was a grant applicant, participated in the Steering Committee, and advised on trial delivery by ambulance services.
- GV was independent chair of the Steering Committee.
- JMW was a grant applicant, participated in the Steering Committee, and led adjudication of brain scans.
- NSp was deputy chief investigator, a grant applicant, and participated in the Steering Committee.
- The corresponding author wrote the first draft of the manuscript, and this was edited and commented on by the Writing Committee. All members of the Writing Committee commented on the analyses and drafts of this report and have seen and approved the final version of the report.

LIST OF SUPPLEMENTARY TABLES AND FIGURES

	Title	Page
**Tables**		
I	Adherence and reasons for non-adherence	11
II	In hospital management and treatment	12
III	Neuroimaging findings on admission to hospital and at days 2-4	13
IV	Intracerebral haematoma volume in recent ICH trials	15
V	Relationship between admission imaging characteristics and outcome	16
VI	Functional outcome (modified Rankin scale) by time from onset to randomisation in recent ICH trials	18
**Figures**		
I	Blood pressure profile by treatment group	19
II	Changes in A) Glasgow coma scale and B) Face-Arm-Speech test from baseline by treatment group	20
III	Cumulative case fatality during the 90 days of follow-up	21
IV	Forest plot of outcomes included in the global analysis	22
V	Shift in haematoma size (as maximum length) on admission to hospital by treatment group	23
VI	Shift in haematoma mass effect on admission to hospital by treatment group	24
VII	Box and whisker plot for modified Rankin scale by maximum length of haematoma	25
VIII	Meta-analysis of A) death or dependency, and B) death, by time to recruitment in trials including patients with intracerebral haemorrhage	26

Table I. Adherence and reasons for non-adherence, in confirmed intracerebral haemorrhage. Data are number (%).

	All	GTN	Sham
Participants with data	145	74	71
Adherence (%), received			
First treatment	145 (100)	74 (100)	71 (100)
At least first 2 days of treatment [a]	88 (61)	45 (61)	43 (61)
All 4 days treatment	70 (48)	35 (47)	35 (49)
Reasons for non-adherence (%) [b]			
Non-stroke diagnosis initially	0 (0)	0 (0)	0 (0)
Serious adverse event	1 (1)	0 (0)	1 (1)
Adverse event (not an SAE)	0 (0)	0 (0)	0 (0)
Discharged before day 2	1 (1)	1 (1)	0 (0)
Participant/proxy refused patch	0 (0)	0 (0)	0 (0)
Medical decision to stop treatment	15 (10)	9 (12)	6 (8)
Procedural error	7 (5)	3 (4)	4 (6)
Trial medication missing/not available	6 (4)	4 (5)	2 (3)
Died	14 (10)	7 (9)	7 (10)
Others	12 (8)	4 (5)	8 (11)

[a] Patients receiving at least the first two days of treatment are considered to have been adherent to treatment
[b] Reasons for non-adherence are not mutually exclusive

Table II. In hospital management and treatment for 145 participants with a final diagnosis of intracerebral haemorrhage by treatment group – glyceryl trinitrate (GTN) versus sham

Outcome (%)	N	GTN	Sham	aOR/aMD (95% CI)
Antihypertensive use	141	44 (62)	53 (76)	0.52 (0.23, 1.15)
Randomisation to BP treatment (mins)	91	262 [85, 1624]	321 [95, 1218]	188 (-184, 559)
Alpha-receptor antagonist (%)	141	1 (1.4)	1 (1.4)	1.47 (0.07, 31.63)
ACE-I (%)	141	12 (16.9)	13 (18.6)	0.90 (0.35, 2.33)
ARA (%)	141	1 (1.4)	6 (8.6)	0.17 (0.02, 1.48)
Beta-receptor antagonist (%)	141	28 (39.4)	30 (42.9)	0.76 (0.35, 1.64)
Labetalol	141	25 (35)	19 (27)	1.37 (0.60, 3.14)
Calcium-channel blocker (%)	141	21 (29.6)	24 (34.3)	0.88 (0.41, 1.87)
Centrally acting agent (%)	141	0 (0)	1 (1.4)	-
Diuretic (%)	141	3 (4.2)	5 (7.1)	0.61 (0.13, 2.79)
Nitrate (%)	141	14 (19.7)	22 (31.4)	0.53 (0.24, 1.21)
Other antihypertensive (%)	141	0 (0)	1 (1.4)	-
Hemicraniectomy	141	2 (2.8)	1 (1.4)	1.37 (0.06, 29.97)
Other surgery	141	4 (5.6)	3 (4.3)	1.34 (0.24, 7.62)
Neurosurgical Unit	143	5 (6.8)	4 (5.7)	1.07 (0.24, 4.77)
Intensive care	143	13 (17.8)	8 (11.4)	1.71 (0.61, 4.85)
Ventilation	143	12 (16.4)	4 (5.7)	3.62 (1.00, 13.1)
Acute Stroke Unit	145	58 (78.4)	58 (81.7)	0.70 (0.30, 1.64)
SRU	145	24 (32.4)	24 (33.8)	0.81 (0.37, 1.78)
Physiotherapy	143	41 (56.9)	56 (78.9)	0.28 (0.12, 0.65)
Occupational therapy	143	41 (56.9)	47 (66.2)	0.55 (0.26, 1.17)
Speech therapy	143	40 (55.6)	50 (70.4)	0.36 (0.17, 0.79)

ACE-I: angiotensin-converting enzyme inhibitor; aOR: adjusted odds ratio; ARA: angiotensin-II receptor antagonist; CI: confidence intervals; SRU: Stroke Rehabilitation Unit

Table III. Neuroimaging findings on admission to hospital (post-treatment) and at days 2-4. Data are number (%), median [interquartile quartile range] or mean (standard deviation), and adjusted common odds ratio (acOR), adjusted odds ratio (aOR), or adjusted difference in means (aDIM), with 95% confidence intervals. Comparison by binary logistic regression (BLR), Cox proportional hazards regression (Cox), ordinal logistic regression (OLR), or multiple linear regression (MLR), with adjustment for age, sex, pre-morbid mRS, FAST, pre-treatment systolic BP, and time to randomisation (unless stated).

	Admission			acOR/aOR/aDIM (95% CI)	Day 2-4			acOR/aOR/aDIM (95% CI)
Outcome	N	GTN N=74	Sham N=71		N	GTN N=54	Sham N=46	
Brain scan type								
CT	144	73 (100)	71 (100)	-	97	52 (98)	43 (98)	
MRI	144	0 (0)	0 (0)	-	97	1 (2)	1 (2)	
Onset to scan (hours)	143	2.3 [1.7,2.8]	2.3 [1.9,2.8]	1.15 (0.65, 2.02)	103	29.6 [25.8,41.6]	28.2 [23.3,39.2]	1.40 (0.71, 2.76)
Pre-stroke								
Atrophy	144	67 (92)	67 (94)	0.67 (0.18, 2.47)	-			
PVL	144	41 (56)	33 (46)	1.48 (0.77, 2.85)				
Old stroke(s)	144	45 (62)	46 (65)	0.87 (0.44, 1.72)				
Frailty score [/3]	144	3 [3,4]	3 [3,4]	1.15 (0.63, 2.10)				
SVD score [/2]	144	2 [1,2]	2 [1,2]	1.02 (0.55, 1.88)				
Haematoma								
Location (%)	112			0.89 (0.43, 1.86)	-			
Deep		33 (59)	33 (59)					
Lobar		19 (34)	15 (27)					
Posterior		4 (7)	8 (14)					
Length [1-4] [a]	142	2 [2, 3]	2 [1, 3]	1.95 (1.07, 3.58)	97	2 [1,3]	1 [1,2.5]	2.05 (0.97, 4.34)
Volume (ml)	123	38.4 (33.4)	32.3 (34.9)	6.11 (-5.86, 18.08)	84	45.3 (37.3)	28.4 (29.8)	16.95 (2.47, 31.44)
Expansion (%) [b]	-				83	21 (46)	10 (27)	2.27 (0.90, 5.74)
Shape [/5]	123	3.5 [3,5]	3 [2,4]	1.70 (0.90, 3.21)	84	4 [3,5]	3 [2,4]	2.71 (1.22, 6.01)
Density [/5]	123	2 [1,3]	2 [1,3]	1.41 (0.74, 2.68)	84	2 [2,3]	2 [1,3]	1.79 (0.81, 3.94)
Attenuation (mean)	123	53.7 (7.1)	53.2 (6.5)	0.49 (-1.91, 2.89)	83	54.1 (7.7)	53.9 (7.0)	0.21 (-2.94, 3.36)
Blend sign (%)	123	6 (10)	7 (12)	0.83 (0.26, 2.62)	84	3 (7)	1 (3)	2.58 (0.26, 25.89)
Black hole sign (%)	123	15 (24)	15 (25)	0.98 (0.43, 2.23)	84	4 (9)	0 (0)	-
Island sign (%)	123	12 (19)	7 (12)	1.85 (0.68, 5.08)	84	6 (13)	1 (3)	5.55 (0.64, 48.30)

Hypodensity sign (%)	123	23 (37)	20 (33)	1.21 (0.58, 2.54)	84	9 (20)	8 (21)	0.91 (0.31, 2.65)
Swirl sign (%)	123	8 (13)	9 (15)	0.86 (0.31, 2.39)	84	3 (7)	1 (3)	2.58 (0.26, 25.89)
IVH								
IVH (%)	143	32 (44)	25 (35)	1.47 (0.75, 2.89)	97	28 (53)	21 (48)	1.23 (0.55, 2.73)
Volume (ml)	64	14 (16.0)	18 (31.3)	-3.9 (-15.7, 7.8)	48	13.1 (14.9)	10 (13.0)	3.07 (-5.08, 11.23)
Graeb	64	4 [2, 7]	3 [2, 8]	0.95 (0.40, 2.28)	48	4 [3, 6]	3 [2, 7]	1.84 (0.64, 5.32)
Modified Graeb	64	5 [2, 11]	4 [2, 11]	1.06 (0.45, 2.52)	48	4.5 [3, 10]	3 [2, 9]	1.51 (0.53, 4.32)
Mass effect								
Mass effect [/6]	144	2 [2, 4]	2 [2, 2]	2.42 (1.26, 4.68)	97	2 [2,3]	2 [2,2]	1.60 (0.74, 3.49)
PHO	122	14.3 (13.3)	16 (24.6)	-1.74 (-8.67, 5.19)	84	26.7 (19.3)	15.2 (16.6)	11.50 (3.80, 19.20)
Midline shift (%)	123	26 (42)	18 (30)	1.73 (0.82, 3.64)	84	26 (57)	9 (24)	4.19 (1.62, 10.81)
Hydrocephalus (%)	143	35 (48.6)	25 (35.2)	1.74 (0.89, 3.41)	97	34 (64.2)	20 (45.5)	2.15 (0.95, 4.86)
Subdural (%)	143	2 (2.8)	3 (4.2)	0.65 (0.10, 4.00)	97	2 (3.8)	3 (6.8)	0.54 (0.09, 3.36)
ICH CTA								
Spot sign (%)	17	1 (10.0)	1 (14.3)	0.67 (0.03, 12.84)	4	1 (50.0)	0 (0.0)	-

CT: computerised tomography; ICH: intracerebral haemorrhage; IVH: intraventricular haemorrhage; MRI: magnetic resonance imaging; PHO: perihaematomal oedema; PVL: periventricular lucency; SVD: small vessel disease

[a] Haematoma length (cm): 1: <3, 2: 3-5, 3: 5-8, 4: >8.

[b] Expansion is absolute increase in haematoma volume of more than 6 mL or a relative growth of greater than 33% between hospital admission and day 2-4

Table IV. Intracerebral haematoma volume, mean (standard deviation), at hospital admission in recent trials: ordered by haematoma size in control group

Control (ml)	Active (ml)	Trial	Intervention
7.2 (-)	8.3 (-)	CLEAR-3 [17]	Alteplase
7.9 (-)	7.2 (-)	CLEAR-IVH [18]	Alteplase
12.5 (26.8)	14.1 (26.5)	TICH-2 [19]	Tranexamic acid
13.3 (17.7)	13.2 (15.3)	ENOS-ICH [1]	Glyceryl trinitrate
14.2 (14.5)	12.7 (11.6)	INTERACT [20]	Blood pressure lowering
14.3 (-)	27.0 (-)	TICH [21]	Tranexamic acid
15.1 (14.9)	15.7 (15.7)	INTERACT-2 [13]	Blood pressure lowering
22.0 (24.0)	24.0 (26.0)	FAST-2 [22]	Factor VIIa
24.0 (22.0)	24.0 (26.0)	FAST [23]	Factor VIIa
32.3 (34.9)	38.4 (33.4)	RIGHT-2 [5, 6, 24, 25]	Glyceryl trinitrate
37 (-)	40 (-)	STICH [26]	Surgery
41.0 (22.9)	41.4 (21.2)	STICH-2 [27]	Surgery
43.1 (15.3)	48.2 (19.6)	MISTIE-2 [28]	Surgery/alteplase
45.3 (24.4)	42.7 (24.1)	MISTIE-3 [29]	Surgery/alteplase
-	47.2 (19.2)	MISTIE [30]	Surgery/alteplase

Table V. Relationship between imaging characteristics on admission and death, and death or dependency, at day 90. Data are odds ratio (OR) or hazard ratio (HR). Analyses using binary logistic regression, Cox proportional hazards regression or ordinal logistic regression.

	Haematoma expansion [a] OR (95% CI)	p	P [b]	Death HR (95% CI)	p	p†	mRS OR (95% CI)	p	p†
Patients with data	145			145			145		
Patients with outcome	83			144			142		
Pre-stroke									
Atrophy	0.58 (0.08, 4.34)	0.60	0.097	0.50 (0.22, 1.17)	0.11	0.96	0.51 (0.14, 1.88)	0.31	1.00
PVL	0.94 (0.39, 2.28)	0.89	0.003	1.22 (0.72, 2.05)	0.46	0.94	1.42 (0.78, 2.59)	0.25	0.67
Old stroke	1.17 (0.43, 3.18)	0.76	0.10	0.70 (0.42, 1.19)	0.19	0.098	0.61 (0.33, 1.15)	0.13	0.13
Frailty score	0.94 (0.56, 1.58)	0.82	0.002	0.91 (0.67, 1.22)	0.51	0.42	0.95 (0.67, 1.33)	0.75	0.39
SVD score	1.21 (0.66, 2.21)	0.54	0.005	1.06 (0.75, 1.50)	0.75	0.29	1.08 (0.72, 1.63)	0.71	0.097
Haematoma									
Length	1.43 (0.87, 2.37)	0.16	0.71	1.99 (1.51, 2.62)	<0.001	0.87	2.15 (1.53, 3.00)	<0.001	0.67
Volume	1.01 (1.00, 1.03)	0.15	0.43	1.02 (1.01, 1.02)	<0.001	0.25	1.03 (1.01, 1.04)	<0.001	0.67
Shape	0.93 (0.66, 1.32)	0.69	0.022	1.35 (1.07, 1.70)	0.011	0.031	1.32 (1.02, 1.70)	0.032	0.13
Density	2.09 (1.27, 3.45)	0.004	0.78	2.14 (1.60, 2.85)	<0.001	0.56	2.20 (1.54, 3.13)	<0.001	0.54
Attenuation	1.02 (0.95, 1.10)	0.54	0.54	1.00 (0.96, 1.04)	0.96	0.74	0.99 (0.94, 1.04)	0.73	0.62
Blend sign	1.29 (0.27, 6.17)	0.75	0.93	1.45 (0.65, 3.24)	0.36	0.075	2.54 (0.82, 7.92)	0.11	0.081
Black hole sign	1.30 (0.46, 3.68)	0.63	0.56	1.74 (0.96, 3.18)	0.070	0.64	2.04 (0.94, 4.44)	0.071	0.60
Island sign	6.68 (1.65, 27.10)	0.008	0.021	2.32 (1.21, 4.47)	0.012	0.73	3.57 (1.28, 9.93)	0.015	0.80
Hypodensity sign	1.96 (0.77, 5.02)	0.16	0.44	2.15 (1.22, 3.79)	0.008	0.26	2.33 (1.14, 4.77)	0.020	0.43
Swirl sign	0.59 (0.14, 2.41)	0.46	0.76	1.85 (0.92, 3.72)	0.084	0.57	4.00 (1.45, 11.09)	0.008	0.36
IVH									
IVH [c]	0.41 (0.16, 1.03)	0.059	0.34	2.06 (1.22, 3.47)	0.007	0.14	2.16 (1.15, 4.06)	0.017	0.31
Volume [c]	0.94 (0.87, 1.02)	0.15	0.15	1.01 (1.00, 1.02)	0.15	0.18	1.03 (1.00, 1.07)	0.059	0.016
Graeb [c]	0.72 (0.51, 1.02)	0.064	0.15	1.06 (0.94, 1.20)	0.33	0.13	1.10 (0.94, 1.28)	0.25	0.078
Modified Graeb [c]	0.81 (0.64, 1.03)	0.080	0.14	1.02 (0.96, 1.08)	0.55	0.17	1.04 (0.96, 1.12)	0.31	0.073
Mass effect									
Mass effect	1.39 (0.97, 1.99)	0.076	0.78	1.44 (1.25, 1.66)	<0.001	0.72	1.62 (1.30, 2.01)	<0.001	0.64
PHO	1.02 (0.98, 1.07)	0.23	0.17	1.02 (1.01, 1.03)	<0.001	0.50	1.06 (1.03, 1.09)	<0.001	0.29
Midline shift	1.24 (0.48, 3.18)	0.66	0.40	1.90 (1.08, 3.36)	0.026	0.086	2.88 (1.43, 5.82)	0.003	0.26
Hydrocephalus	0.71 (0.27, 1.85)	0.48	0.99	4.10 (2.34, 7.18)	<0.001	0.001	5.21 (2.63, 10.31)	<0.001	0.009

IVH: intraventricular haemorrhage; PHO: perihaematomal oedema; PVL: periventricular lucency; SVD: small vessel disease
Haematoma expansion defined as an absolute increase of more than 6 mL or a relative growth of greater than 33%.

[a] Expansion is absolute increase in haematoma volume of more than 6 mL or a relative growth of greater than 33% between hospital admission and day 2-4
[b] p: p for interaction with GTN vs sham
[c] No IVH: assigned 0 value

Table VI. Functional outcome (modified Rankin scale) by time from onset to randomisation in recent intracerebral haemorrhage trials involving a vasodilator.

Trial	OTR (mins)	N	Outcome	mRS
RIGHT-2	74	142	Negative	1.87 (0.98, 3.57)
ATACH-2 [31]	182	961	Neutral	1.02 (0.83, 1.25)
INTERACT-2 [13]	222	2894	Positive	0.87 (0.77, 1.00)
ENOS-early [a] [14]	280	61	Positive	0.22 (0.07, 0.69)
ENOS-rest [b] [15]	1601	568	Neutral	1.16 (0.86, 1.57)

mRS: modified Rankin scale; OTR: onset to randomisation
[a] ENOS participants randomised within 6 hours of onset
[b] ENOS participants randomised after 6 hours of onset
The RIGHT trial is not shown since it only enrolled 6 ICH patients [8]

Figure I. Blood pressure profile by treatment group – glyceryl trinitrate (GTN) versus sham.

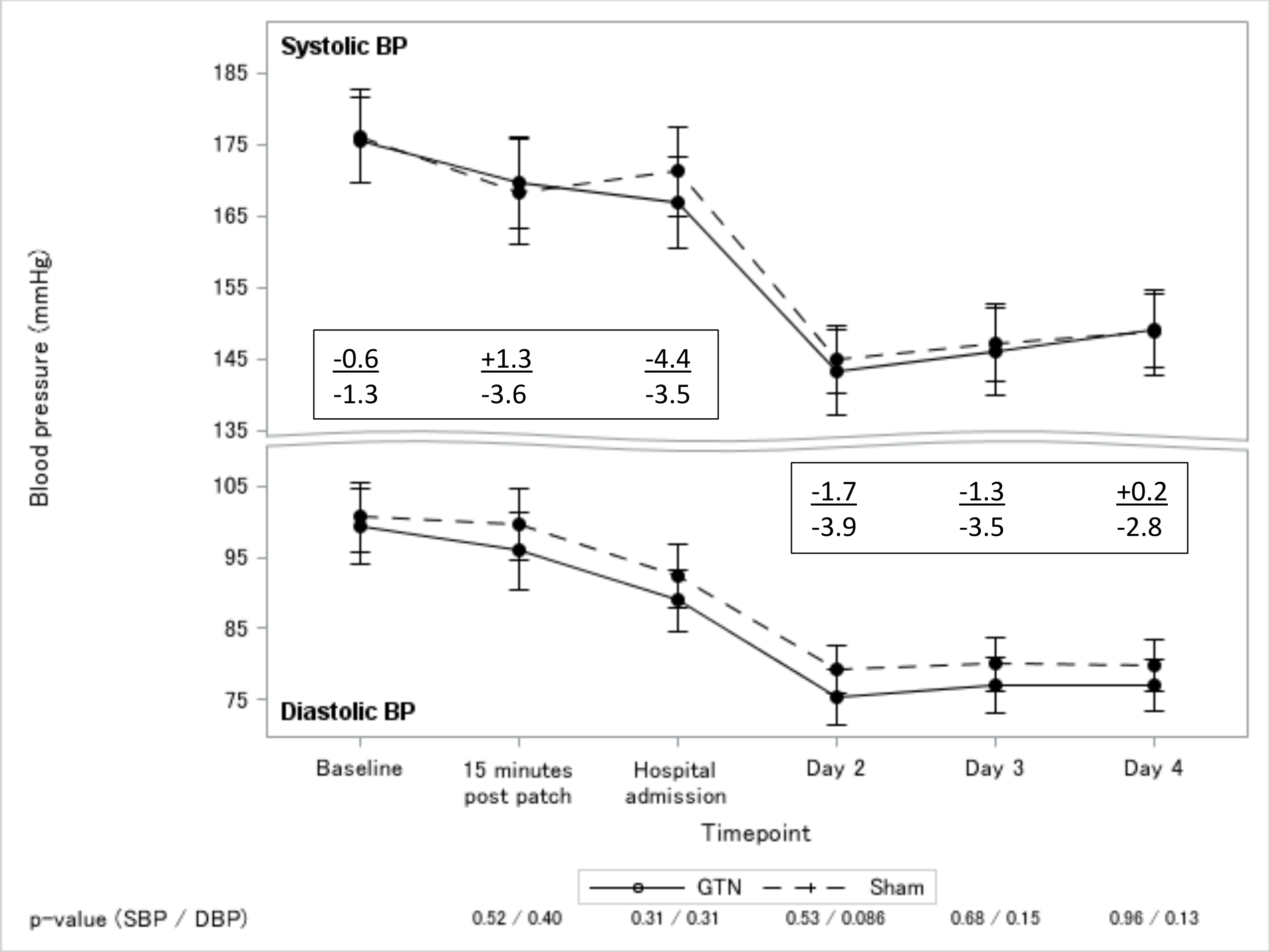

Figure II. Changes in Glasgow coma scale and Face-Arm-Speech test from baseline in ambulance by treatment group – glyceryl trinitrate (GTN) versus sham.

A) Glasgow coma scale (GCS) with Bonferroni corrected ANCOVA

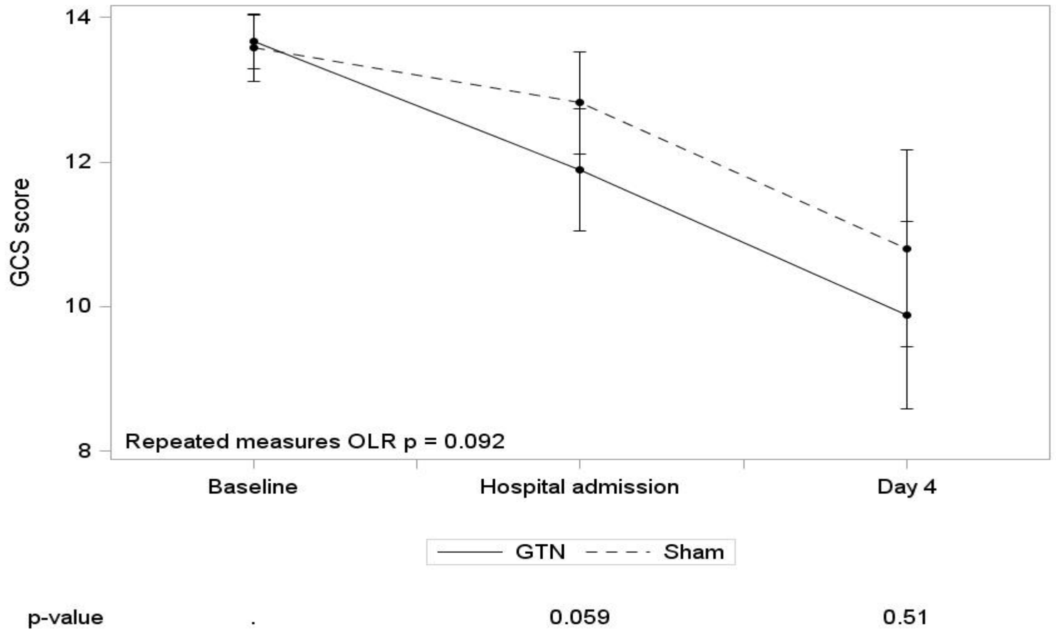

B) Face-Arm-Speech test with Bonferroni corrected ANCOVA

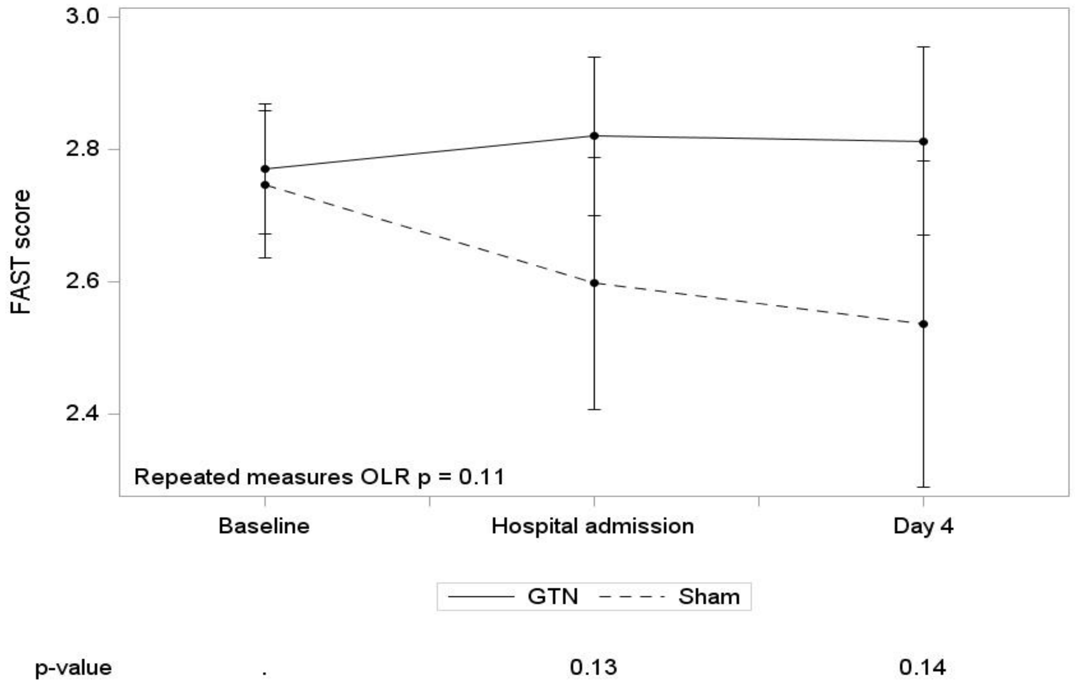

Figure III. Cumulative case fatality during the 90 days of follow-up after randomisation by treatment group – glyceryl trinitrate (GTN) versus sham. Comparison of GTN versus sham by Cox regression with adjustment for age, sex, pre-morbid mRS, FAST, pre-treatment systolic BP, and time to treatment.

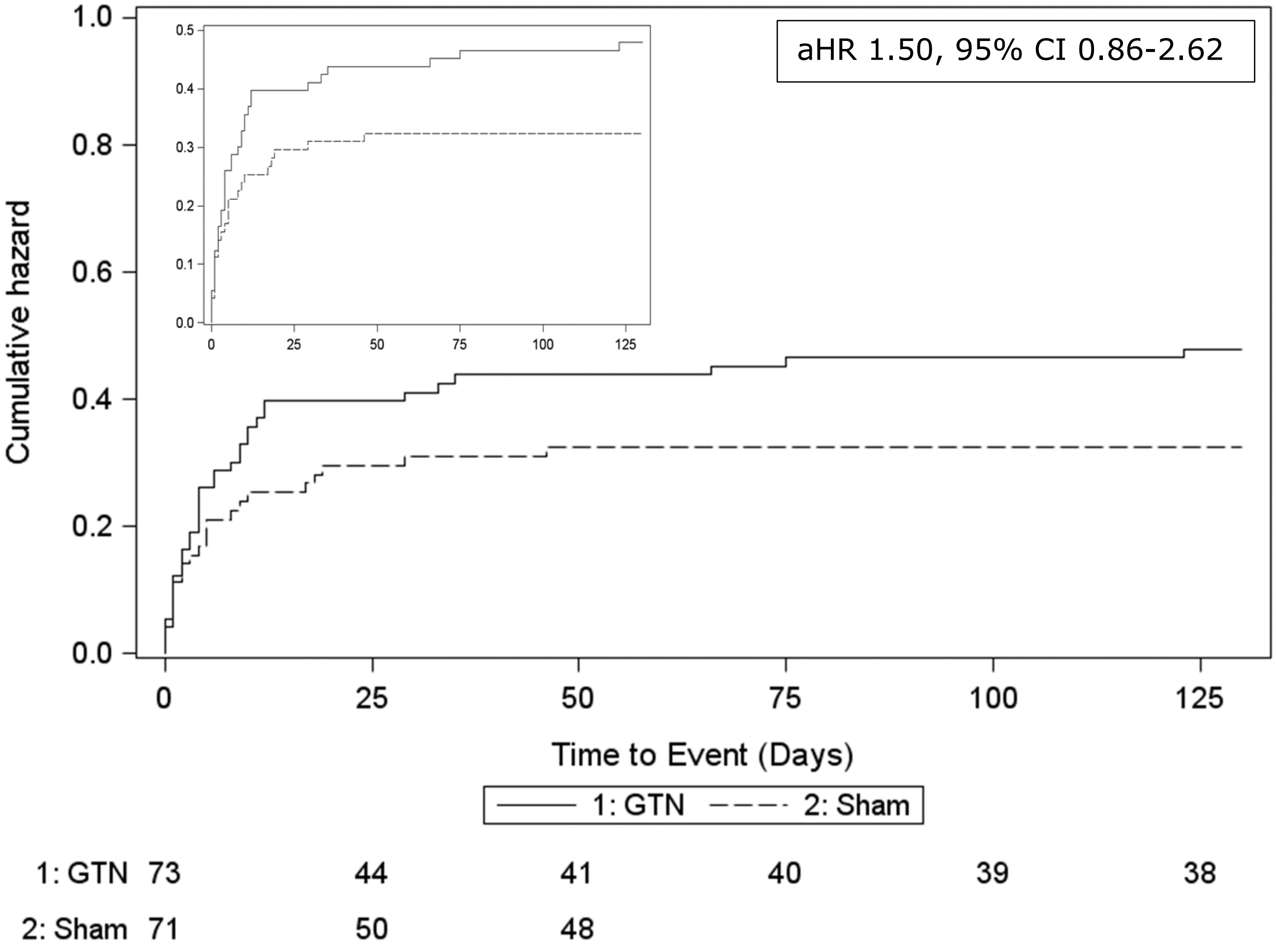

Figure IV. Forest plot of outcomes included in the global analysis (modified Rankin scale, Barthel index, Euro-Qol-5D, Zung depression scale, telephone interview cognition scale-modified – Wei-Lachin test) in participants with intracerebral haemorrhage.

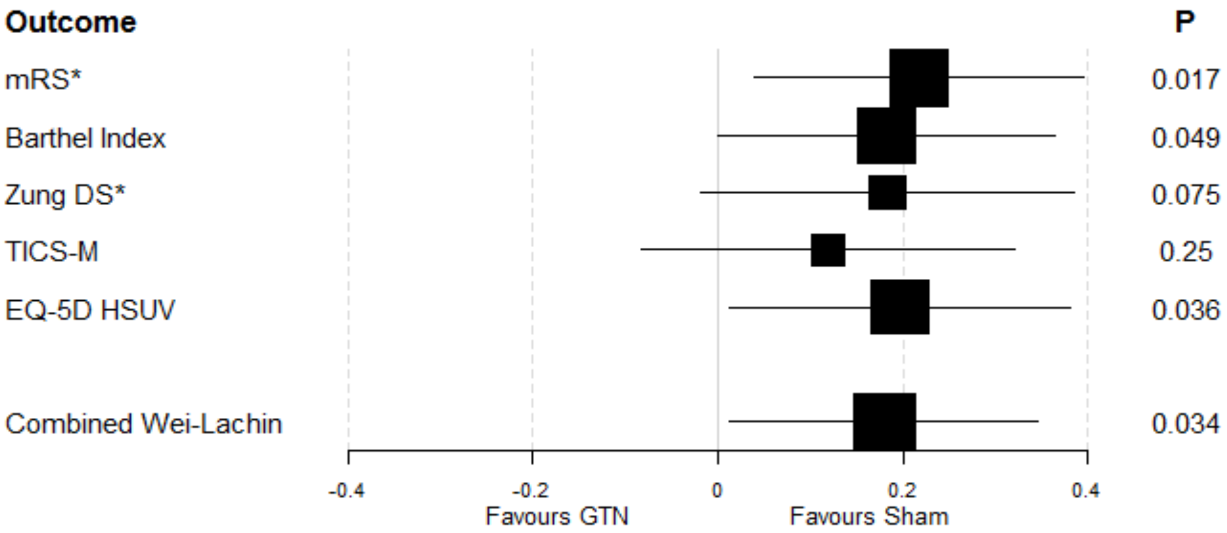

Figure V. Shift in haematoma size (as maximum length) on admission to hospital by treatment group – glyceryl trinitrate (GTN) versus sham.

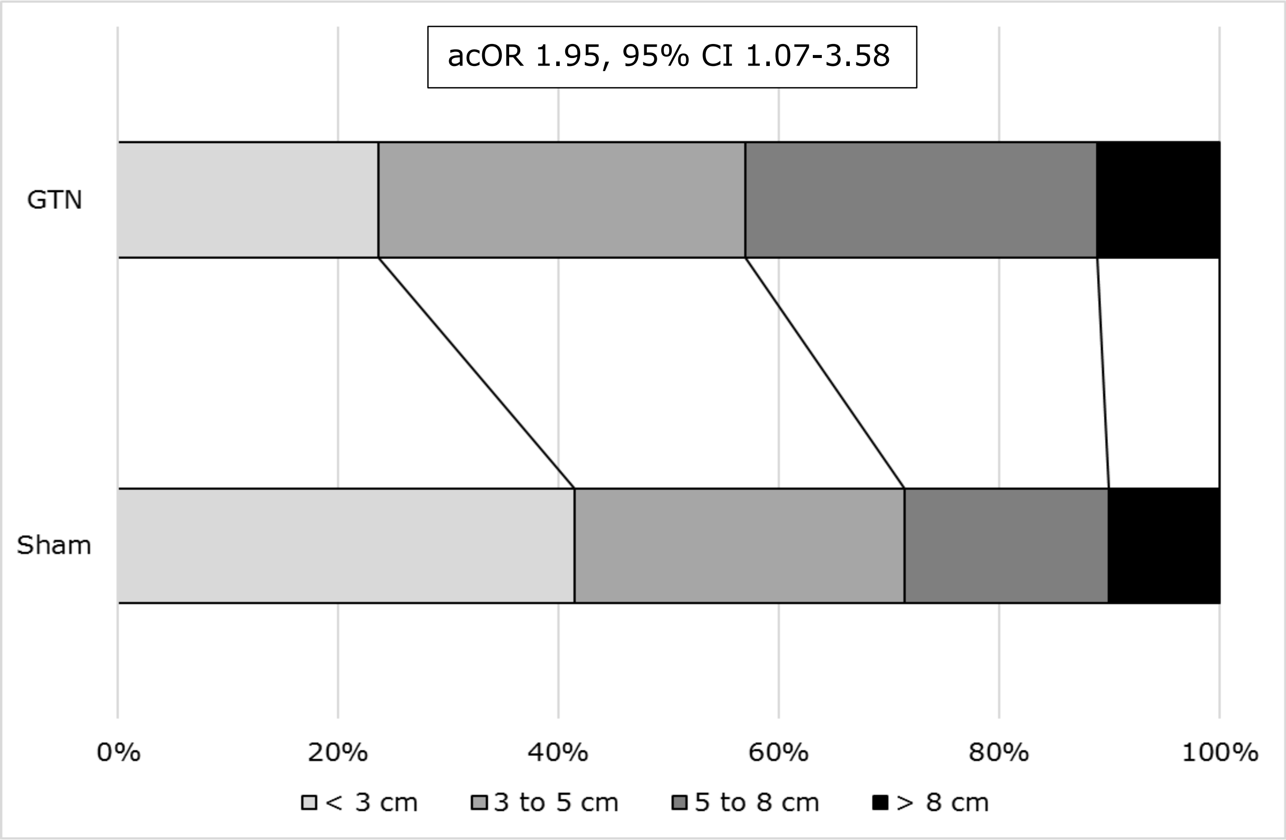

Figure VI. Shift in haematoma mass effect on admission to hospital by treatment group – glyceryl trinitrate (GTN) versus sham.

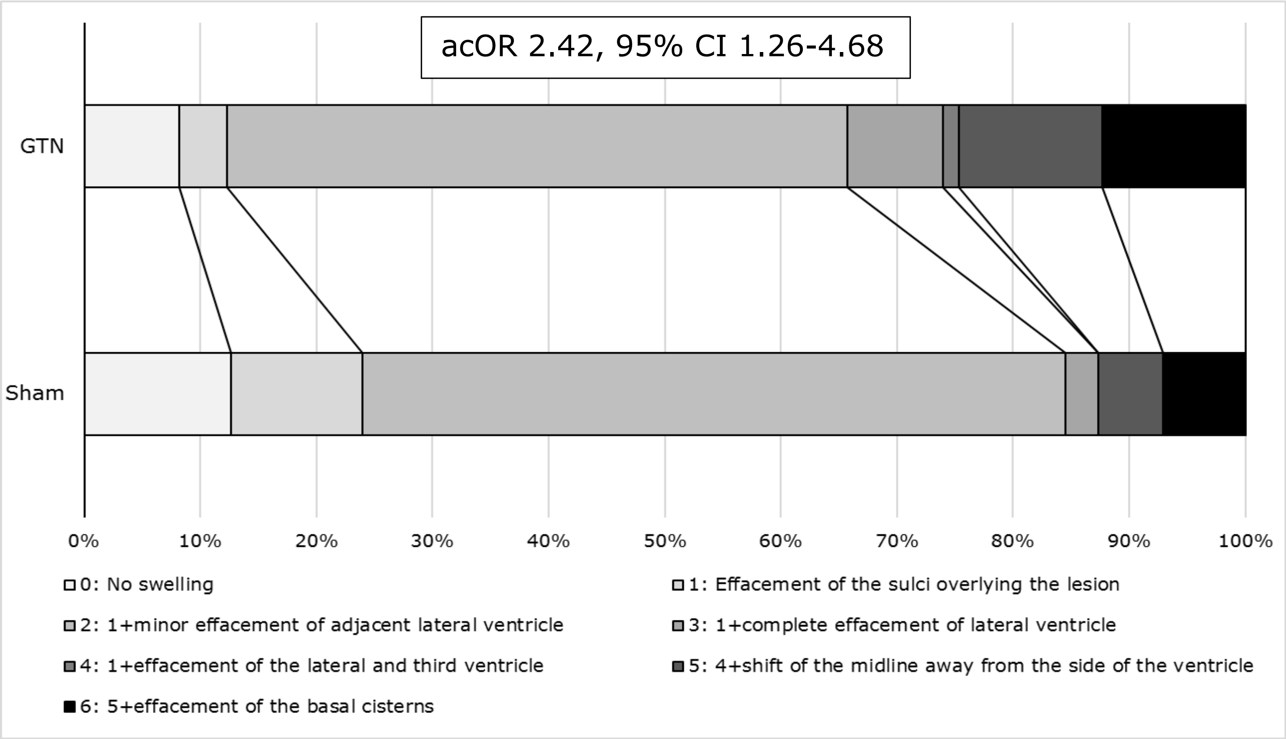

Figure VII. Box and whisker plot for modified Rankin scale by maximum length of haematoma (<3, 3-5, 5-8 and >8 cm).

Overall Statistics				
Min	1	1	1	2
Mean	3.840909	4.636364	5.083333	5.4
Max	6	6	6	6
Std Dev	1.49329	1.643747	1.273353	1.242118

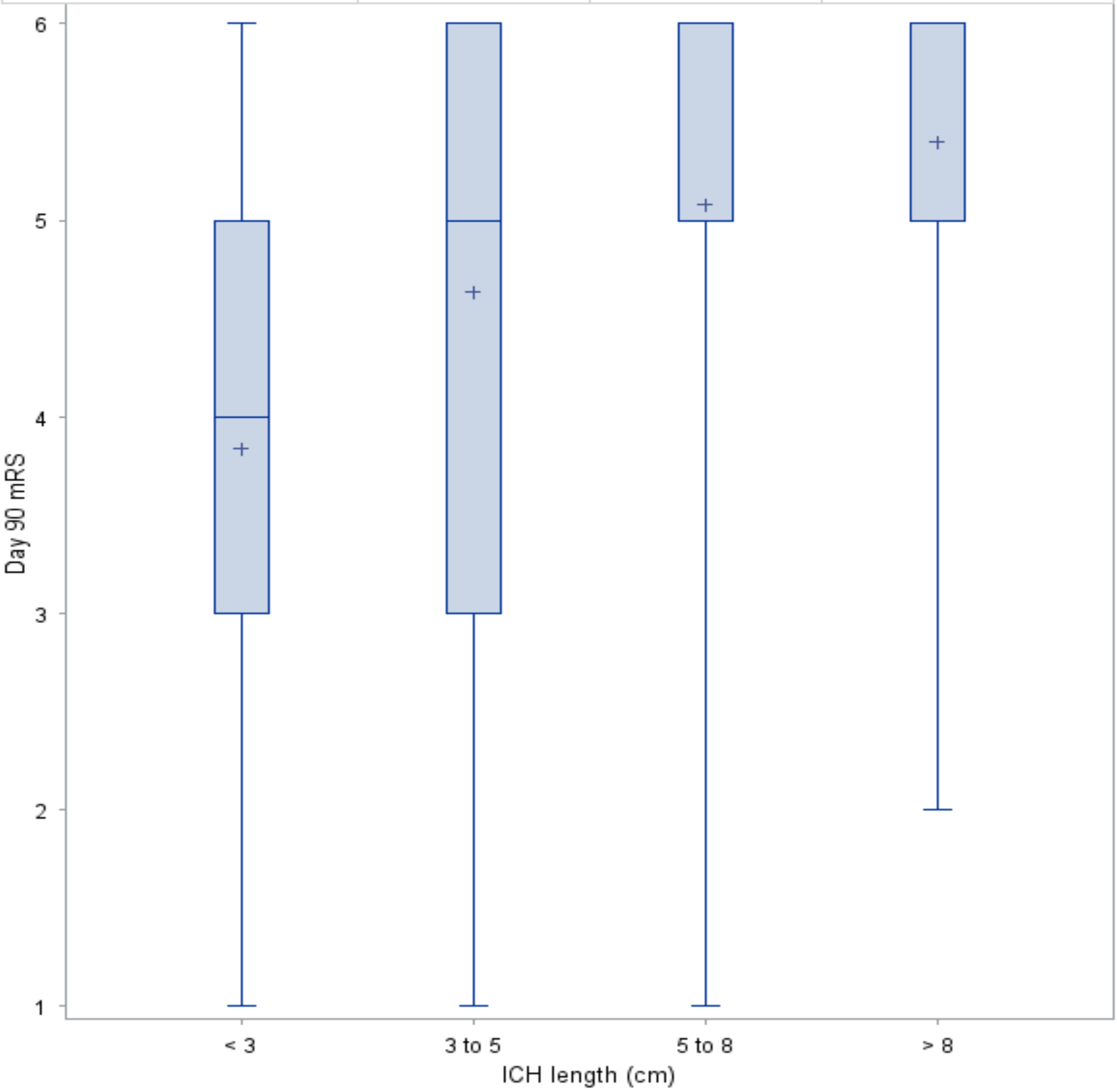

Figure VIII. Meta-analysis of A) death or dependency, and B) death, by time to recruitment in trials including patients with intracerebral haemorrhage.

A) Death or dependency, modified Rankin Scale >2

Study or Subgroup	GTN Events	GTN Total	Control Events	Control Total	Weight	Odds Ratio M-H, Random, 95% CI	Year
**1.1.1 0-2 hours**							
RIGHT 2013	2	4	1	1	11.0%	0.33 [0.01, 12.82]	2013
ENOS-early 2015	1	1	0	0		Not estimable	2015
RIGHT-2 2018	52	54	49	59	30.3%	5.31 [1.11, 25.44]	2018
**Subtotal (95% CI)**		**59**		**60**	**41.4%**	**2.22 [0.18, 27.57]**	
Total events	55		50				
Heterogeneity: $Tau^2 = 1.78$; $Chi^2 = 1.87$, df = 1 (P = 0.17); $I^2 = 46\%$							
Test for overall effect: Z = 0.62 (P = 0.54)							
**1.1.2 2-6 hours**							
RIGHT 2013	1	1	0	0		Not estimable	2013
ENOS-early 2015	17	28	21	30	38.2%	0.66 [0.22, 1.97]	2015
RIGHT-2 2018	13	17	11	12	20.5%	0.30 [0.03, 3.05]	2018
**Subtotal (95% CI)**		**46**		**42**	**58.6%**	**0.57 [0.21, 1.54]**	
Total events	31		32				
Heterogeneity: $Tau^2 = 0.00$; $Chi^2 = 0.38$, df = 1 (P = 0.54); $I^2 = 0\%$							
Test for overall effect: Z = 1.11 (P = 0.27)							
**Total (95% CI)**		**105**		**102**	**100.0%**	**0.98 [0.25, 3.86]**	
Total events	86		82				
Heterogeneity: $Tau^2 = 0.97$; $Chi^2 = 6.37$, df = 3 (P = 0.09); $I^2 = 53\%$							
Test for overall effect: Z = 0.03 (P = 0.98)							
Test for subgroup differences: $Chi^2 = 0.96$, df = 1 (P = 0.33), $I^2 = 0\%$							

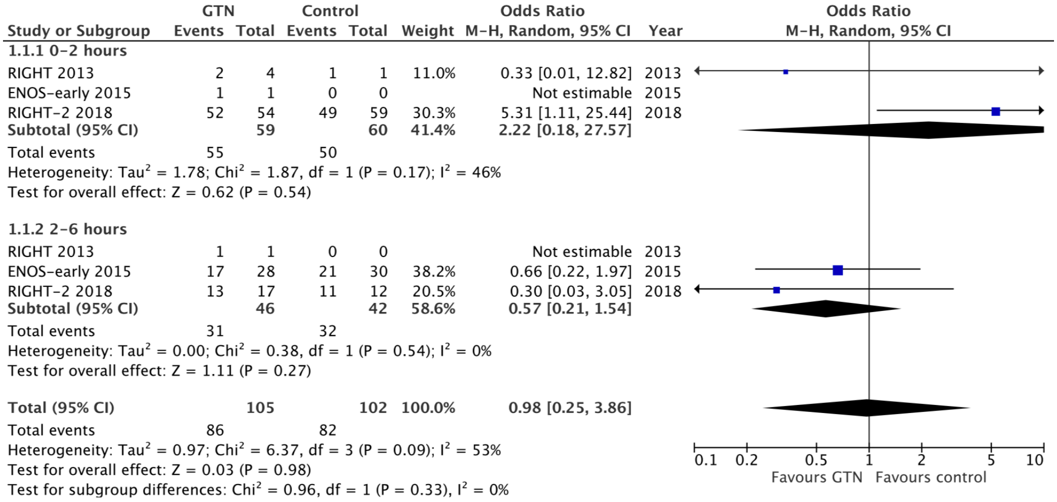

B) Death

Study or Subgroup	NO donor Events	NO donor Total	Control Events	Control Total	Weight	Odds Ratio M-H, Random, 95% CI
**1.9.1 0-2 hours**						
ENOS-early 2015	0	1	1	2	10.1%	0.33 [0.01, 16.80]
RIGHT 2013	1	4	1	1	10.9%	0.14 [0.00, 5.95]
RIGHT-2 2018	30	54	19	59	30.7%	2.63 [1.22, 5.66]
**Subtotal (95% CI)**		**59**		**62**	**51.7%**	**1.10 [0.18, 6.84]**
Total events	31		21			
Heterogeneity: $Tau^2 = 1.19$; $Chi^2 = 3.18$, df = 2 (P = 0.20); $I^2 = 37\%$						
Test for overall effect: Z = 0.10 (P = 0.92)						
**1.9.2 2-6 hours**						
ENOS-early 2015	2	28	11	30	24.0%	0.13 [0.03, 0.67]
RIGHT 2013	1	1	0	0		Not estimable
RIGHT-2 2018	5	19	4	12	24.3%	0.71 [0.15, 3.45]
**Subtotal (95% CI)**		**48**		**42**	**48.3%**	**0.31 [0.06, 1.63]**
Total events	8		15			
Heterogeneity: $Tau^2 = 0.77$; $Chi^2 = 2.15$, df = 1 (P = 0.14); $I^2 = 54\%$						
Test for overall effect: Z = 1.38 (P = 0.17)						
**Total (95% CI)**		**107**		**104**	**100.0%**	**0.55 [0.12, 2.47]**
Total events	39		36			
Heterogeneity: $Tau^2 = 1.75$; $Chi^2 = 13.40$, df = 4 (P = 0.009); $I^2 = 70\%$						
Test for overall effect: Z = 0.78 (P = 0.44)						
Test for subgroup differences: $Chi^2 = 1.00$, df = 1 (P = 0.32), $I^2 = 0\%$						

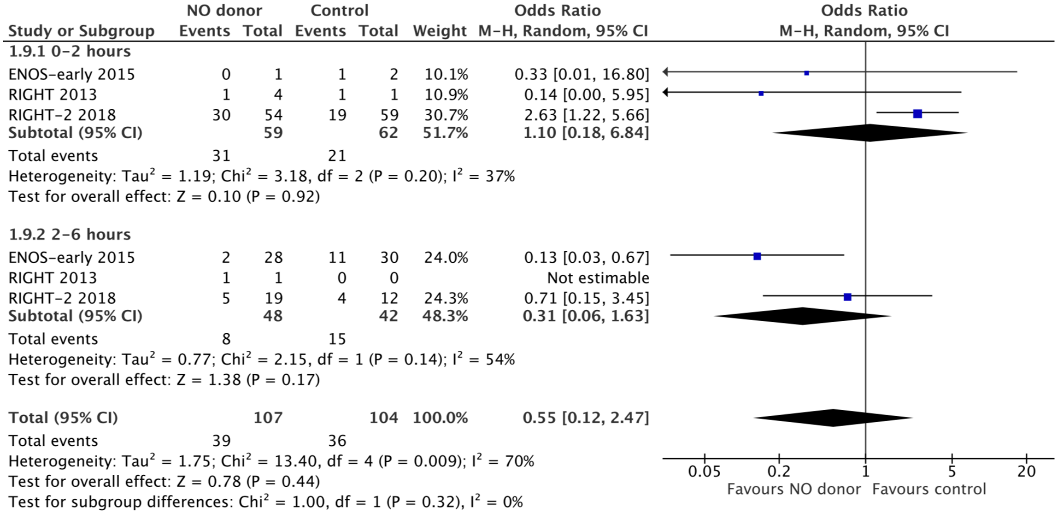

SUPPLEMENTAL REFERENCES

1. Krishnan K, Mukhtar SF, Lingard J, Houlton A, Walker E, Jones T, et al. Performance characteristics of methods for quantifying spontaneous intracerebral haemorrhage: Data from the efficacy of nitric oxide in stroke (enos) trial. *Journal of Neurology, Neurosurgery & Psychiatry*. 2015:jnnp-2014-309845
2. IST-3 collaborative group. Association between brain imaging signs, early and late outcomes, and response to intravenous alteplase after acute ischaemic stroke in the third international stroke trial (ist-3): Secondary analysis of a randomised controlled trial. *The Lancet. Neurology.* 2015;14:485-496
3. Zhang D, Chen J, Xue Q, Du B, Li Y, Chen T, et al. Heterogeneity signs on noncontrast computed tomography predict hematoma expansion after intracerebral hemorrhage: A meta-analysis. *Biomed Research International*. 2018;6038193
4. Wada R, Aviv RI, Fox AJ, Sahlas DJ, Gladstone DJ, Tomlinson G, et al. Ct angiography "spot sign" predicts hematoma expansion in acute intracerebral hemorrhage. *Stroke*. 2007;38:1257-1262
5. Scutt P, Appleton JP, Dixon M, Woodhouse L, Sprigg N, Wardlaw JM, et al. Statistical analysis plan for the 'rapid intervention with glyceryl trinitrate in hypertensive stroke trial-2 (right-2)'. *European Stroke Journal*. 2018;3:193-196
6. RIGHT-2 Investigators. Prehospital transdermal glyceryl trinitrate in patients with ultra-acute presumed stroke (right-2): An ambulance-based, randomised, sham-controlled, blinded, phase 3 trial. *Lancet*. 2019;393:1009-1020
7. Gray LJ, Bath PM, Collier T. Should stroke trials adjust functional outcome for baseline prognostic factors? *Stroke*. 2009;40:888-894
8. Ankolekar S, Fuller M, Cross I, Renton C, Cox P, Sprigg N, et al. Feasibility of an ambulance-based stroke trial, and safety of glyceryl trinitrate in ultra-acute stroke the rapid intervention with glyceryl trinitrate in hypertensive stroke trial (right, isrctn66434824). *Stroke*. 2013;44:3120-3128
9. Tilley BC, Marler J, Geller NL, Lu M, Legler J, Brott T, et al. Use of a global test for multiple outcomes in stroke trials with application to the national institute of neurological disorders and stroke t-pa stroke trial. *Stroke*. 1996;27:2136-2142
10. Periayah MH, Halim AS, Mat Saad AZ. Mechanism action of platelets and crucial blood coagulation pathways in hemostasis. *Int J Hematol Oncol Stem Cell Res*. 2017;11:319-327
11. Bath PM, Appleton JP, Krishnan K, Sprigg N. Blood pressure in acute stroke: To treat or not to treat: That is still the question. *Stroke*. 2018;49:1784-1790
12. Rodrigues MA, Samarasekera N, Lerpiniere C, Humphreys C, McCarron MO, White PM, et al. The edinburgh ct and genetic diagnostic criteria for lobar intracerebral haemorrhage associated with cerebral amyloid angiopathy: Model development and diagnostic test accuracy study. *Lancet Neurol*. 2018;17:232-240
13. Anderson CS, Heeley E, Huang Y, Wang J, Stapf C, Delcourt C, et al. Rapid blood-pressure lowering in patients with acute intracerebral hemorrhage. *N Engl J Med*. 2013;368:2355-2365
14. Woodhouse L, Scutt P, Krishnan K, Berge E, Gommans J, Ntaios G, et al. Effect of hyperacute administration (within 6 hours) of transdermal glyceryl trinitrate, a nitric oxide donor, on outcome after stroke: Subgroup analysis of the efficacy of nitric oxide in stroke (enos) trial. *Stroke*. 2015;46:3194-3201
15. Bath PM, Woodhouse L, Scutt P, Krishnan K, Wardlaw JM, Bereczki D, et al. Efficacy of nitric oxide, with or without continuing antihypertensive treatment,

for management of high blood pressure in acute stroke (enos): A partial-factorial randomised controlled trial. *Lancet*. 2015;385:617-628

16. The AVERT Trial Collaboration group. Efficacy and safety of very early mobilisation within 24 h of stroke onset (avert): A randomised controlled trial. *Lancet*. 2015;386:46-55
17. Hanley DF, Lane K, McBee N, Ziai W, Tuhrim S, Lees KR, et al. Thrombolytic removal of intraventricular haemorrhage in treatment of severe stroke: Results of the randomised, multicentre, multiregion, placebo-controlled clear iii trial. *Lancet*. 2017;389:603-611
18. Naff N, Williams MA, Keyl PM, Tuhrim S, Bullock MR, Mayer SA, et al. Low-dose recombinant tissue-type plasminogen activator enhances clot resolution in brain hemorrhage the intraventricular hemorrhage thrombolysis trial. *Stroke*. 2011;42:3009-3016
19. Sprigg N, Flaherty K, Appleton JP, Al-Shahi Salman R, Bereczki D, Beridze M, et al. Tranexamic acid for hyperacute primary intracerebral haemorrhage (tich-2): An international randomised, placebo-controlled, phase 3 superiority trial. *Lancet*. 2018;391:2107-2115
20. Anderson CS, Huang Y, Wang JG, Arima H, Neal B, Peng B, et al. Intensive blood pressure reduction in acute cerebral haemorrhage trial (interact): A randomised pilot trial. *Lancet Neurol*. 2008;7:391-399
21. Sprigg N, Renton C, Dineen RA, Kwong Y, Bath PMW. Tranexamic acid for spontaneous intracerebral haemorrhage (tich): A randomised controlled pilot trial. *Journal of Stroke and cerebrovascular diseases*. 2013;23:1312-1318
22. Mayer SA, Brun NC, Begtrup K, Broderick J, Davis S, Diringer MN, et al. Efficacy and safety of recombinant activated factor vii for acute intracerebral hemorrhage. *New England Journal of Medicine*. 2008;358:2127-2137
23. Mayer SA, Brun NC, Begtrup K, Broderick J, Davis S, Diringer MN, et al. Recombinant activated factor vii for acute intracerebral hemorrhage. *New England Journal of Medicine*. 2005;352:777-785
24. Appleton JP, Scutt P, Dixon M, Howard H, Haywood L, Havard D, et al. Ambulance-delivered transdermal glyceryl trinitrate versus sham for ultra-acute stroke: Rationale, design and protocol for the rapid intervention with glyceryl trinitrate in hypertensive stroke trial-2 (right-2) trial (isrctn26986053). *Int J Stroke*. 2019;14:191-206
25. Bath PM, Scutt P, Appleton JP, Dixon M, Woodhouse LJ, Wardlaw JM, et al. Baseline characteristics of the 1149 patients recruited into the rapid intervention with glyceryl trinitrate in hypertensive stroke trial-2 (right-2) randomized controlled trial. *Int J Stroke*. 2019;14:298-305
26. Mendelow AD, Gregson BA, Fernades HM, Murray GD, Teasdale GM, Hope TD, et al. Early surgery versus initial conservative treatment in patients with spontaneour supratentorial intracerebral haematomas in the international surgical trial in intracerebral haemorrhage (stich): A randomised trial. *Lancet*. 2005;365:387-397
27. Mendelow AD, Gregson BA, Rowan EN, Murray GD, Gholkar A, Mitchell PM. Early surgery versus initial conservative treatment in patients with spontaneous supratentorial lobar intracerebral haematomas (stich ii): A randomised trial. *Lancet*. 2013;382:397-408
28. Hanley D, Thompson R, Muschelli J, Rosenblum M, McBee N, Lane K, et al. Safety and efficacy of minimally invasive surgery plus alteplase in intracerebral haemorrhage evacuation (mistie): A randomised, controlled, open-label, phase 2 trial. *Lancet Neurology*. 2016;15:1226-1235
29. Hanley D, Thompson R, Rosenblum M, Yenokyan G, Lane K, McBee N, et al. Efficacy and safety of minimally invasive surgery with thrombolysis in

intracerebral haemorrhage evacuation (mistie iii): A randomised, controlled, open-label, blinded endpoint phase 3 trial. *Lancet*. 2019;393:1021-1032
30. Morgan T, Zuccarello M, Narayan R, Keyl P, Lane K, Hanley D. Preliminary findings of the minimally-invasive surgery plus rtpa for intracerebral hemorrhage evacuation (mistie) clinical trial. *Acta Neurochir Suppl*. 2008:147-151
31. Qureshi AI, Palesch YY, Barsan WG, Hanley DF, Hsu CY, Martin RL, et al. Intensive blood-pressure lowering in patients with acute cerebral hemorrhage. *N Engl J Med*. 2016;375:1033-1043
